# Finasteride Has Regionally Different Effects on Brain Oxidative Stress and Acetylcholinesterase Activity in Acute Thioacetamide-Induced Hepatic Encephalopathy in Rats

**DOI:** 10.1371/journal.pone.0134434

**Published:** 2015-08-04

**Authors:** Dušan Mladenović, Nataša Petronijević, Tihomir Stojković, Milica Velimirović, Gordana Jevtić, Dragan Hrnčić, Tatjana Radosavljević, Aleksandra Rašić-Marković, Nebojša Maksić, Dragan Djuric, Olivera Stanojlović

**Affiliations:** 1 Institute of Pathophysiology "Ljubodrag Buba Mihailovic", Faculty of Medicine, University of Belgrade, Dr Subotica 9, Belgrade, Serbia; 2 Institute of Medical and Clinical Biochemistry, Faculty of Medicine, University of Belgrade, Pasterova 2, Belgrade, Serbia; 3 Institute of Medical Physiology "Richard Burian", Faculty of Medicine, University of Belgrade, Višegradska 26/II, Belgrade, Serbia; 4 Centre for Medical Biochemistry, Clinical Centre of Serbia, Pasterova 2, Belgrade, Serbia; University of Modena and Reggio Emilia, ITALY

## Abstract

Finasteride (FIN) inhibits neurosteroid synthesis and potentially improves the course of hepatic encephalopathy (HE). This study aimed to investigate the effects of FIN on brain oxidative stress and acetylcholinesterase (AchE) activity in acute thioacetamide-induced HE in rats. Male Wistar rats were divided into groups: 1. control; 2. thioacetamide-treated group (TAA; 900 mg/kg); 3. finasteride-treated group (FIN; 150 mg/kg); 4. group treated with FIN and TAA (FIN+TAA). Daily doses of FIN (50 mg/kg) and TAA (300 mg/kg) were administered intraperitoneally during three days and in FIN+TAA group FIN was administered 2h before every dose of TAA. FIN pretreatment prevented TAA-induced rise in malondialdehyde level in the cortex due to restoration of catalase activity and increased expression of superoxide dismutase 1 (SOD1) and induced an increase in malondialdehyde level in the thalamus due to reduction of glutathione peroxidase (GPx) and glutathione reductase (GR) activity. Although FIN pretreatment did not affect malondialdehyde level in hippocampus and caudate nucleus, hippocampal SOD1 expression was higher (p<0.05) and GR activity lower in FIN+TAA vs. TAA group (p<0.05). GPx activity was lower in caudate nucleus in FIN+TAA vs. TAA group (p<0.01). FIN pretreatment prevented TAA-induced rise in AchE activity in the thalamus and caudate nucleus and AchE activity correlates inversely in the thalamus (p<0.05) and positively in caudate nucleus (p<0.01) with malondialdehyde level. FIN has regionally selective effects on oxidative stress and AchE activity in the brain in acute TAA-induced HE in rats. The prooxidant role of FIN in the thalamus may be causally linked with inhibition of AchE.

## Introduction

Acute or chronic liver failure increase blood ammonia level, ultimately leading to the development of neuropsychiatric syndrome known as hepatic encephalopathy (HE) [1]. Oxidative stress and alterations in neurotransmission play important roles in the pathogenesis of HE [2–4].

Oxidative stress is closely interconnected with alterations in neurotransmission in the development of HE. Oxidative injury to the neurons impairs synaptic transmission through oxidation and nitration of key synaptic proteins and contributes to cognitive impairment in HE [5,6]. On the other hand, acute HE is associated with increased synaptic glutamate level [7], that induces neuronal Ca^2+^ influx due to activation of N-methyl-D-aspartate (NMDA) receptors, and ultimately results in mitochondrial permeability transition and increased reactive oxygen species (ROS) production [8,9]. Additionally, Ca^2+^ activates neuronal nitric oxide synthase (nNOS) and NO produced by this enzyme reacts with superoxide anion forming peroxynitrites, reactive nitrogen species [6]. Together with ROS reactive nitrogen species activate nuclear factor kappa B (NF-κB), a transcriptional regulator of inducible NOS (iNOS) gene [5,6], followed by increased production of NO and aggravation of oxidative stress. Other mechanisms of ROS generation include cell swelling [4,6], direct effect of ammonia [10] and effects of proinflammatory cytokines [11].

Disturbances in cholinergic transmission as well as changes in acetylcholinesterase (AchE) activity are associated with HE [12,13]. Modulation of cholinergic transmission may prevent neurodegeneration and attenuate neuroinflammation, thus suggesting that alterations in cholinergic transmission may contribute to oxidative injury in HE [14]. On the other hand, hyperammonemia synergistically with proinflammatory cytokines in acute HE, and with manganese in chronic HE increases neurosteroid synthesis in astrocytes [15–17]. Neurosteroids, allopregnanolone (ALLO) and tetrahydro-deoxycorticosterone (THDOC), potentiate GABAergic transmission through allosteric modulation of GABA_A_ receptor activity and contribute to neuronal inhibition in HE [18]. Central nervous system inhibition by neurosteroids may potentially modulate oxidative stress and cholinergic transmission in the brain, but this effect has not been completely investigated. The possible link between oxidative stress, GABAergic and cholinergic transmission suggests that inhibition of neurosteroid synthesis may modulate oxidative stress and eventually improve the course of HE.

Finasteride (FIN) suppresses neurosteroid synthesis via inhibition of 5α-reductase in astrocytic smooth endoplasmic reticulum [19]. This effect was found to reduce ethanol intake in mice after development of addiction [20], modulate the emotional state [21] and pain transmission [22] as well as to alter seizure development [23]. Additionally, FIN improves the motor and EEG signs ofthioacetamide (TAA)-induced HE as well as morphological changes in the liver through incompletely defined mechanisms [24]. The understanding of these mechanisms may contribute to determination of potential therapeutical effects of FIN in HE. FIN inhibits ALLO synthesis in the brain, but this effect cannot fully explain its neuroprotective effects [25], since FIN also inhibits the synthesis of 5-dihydrotestosterone and other steroids [26]. The effects of FIN on oxidative stress and AchE activity in the brain and the contribution of these effects to the course of HE are not elucidated. Based on this background, the aim of our study was to investigate the effects of FIN on oxidative stress and activity of AchE in various brain regions in acute TAA-induced HE in rats.

## Materials and Methods

### Animals

Experiments were performed on male adult Wistar rats, weighing 170–200 g, that were raised on Military Medical Academy in Belgrade. Animals were kept in individual cages (55x35x30 cm) under standard laboratory conditions (ambient temperature 22 ± 2°C, relative humidity 50%, 12/12 h dark/light cycle with lights turned on at 9:00 AM) with free access to pelleted food and tap water. All experimental procedures were in full compliance with Directive of the European Parliament and of the Council (2010/63/EU) and approved by The Ethical Committee of the University of Belgrade (Permission No 1891/2). All efforts were made to minimize suffering of animals.

All animals were divided into following groups: 1. control, saline and 2-hydroxypropyl-β-cyclodextrin (20% wt/vol)-treated group (n = 8); 2. thioacetamide-treated group, TAA (900 mg/kg; n = 18); 3. finasteride-treated group, FIN (150 mg/kg; n = 8); 4. group treated with finasteride (150 mg/kg) and thioacetamide (900 mg/kg), FIN+TAA (n = 8). Daily doses of TAA (300 mg/kg) and FIN (50 mg/kg) were administered intraperitoneally in three subsequent days and in FIN+TAA group FIN was administered 2 h before every dose of TAA. This dose of TAA was chosen, since in our previous study [27] it has been confirmed to induce severe HE, including hepatic coma. This dose and regimen of FIN administration were selected since FIN improves the course of TAA-induced HE [24]. Before administration, TAA was dissolved in saline in concentration of 100 mg/mL, while FIN was solubilized in 20% wt/vol 2-hydroxypropyl-β-cyclodextrin and administered in a stock concentration of 5 mg/mL (injection volume was 0.01 mL/g animal body weight).

Animals were sacrificed 24 h after treatment and blood for determination of ammonia concentration and samples of dorsolateral frontal cortex (including motor areas), hippocampus, thalamus and caudate nucleus were collected for biochemical analyses as described below. These regions were chosen, since they are involved in motor performance and learning, functions that are dominantly changed in HE [15]. Blood ammonia concentration in the samples collected from the right side of the heart was determined by enzymatic test (BioMerieux Lab., France).

### Biochemical analysis

Animals were sacrificed by decapitation without anesthesia to avoid potential influence of anesthetic drug on the brain oxidative status. After decapitation, the brains were quickly removed and four brain regions (dorsolateral frontal cortex, hippocampus, thalamus and caudate nuclei) were immediately homogenized in cold buffered sucrose medium (0.25M sucrose, 10mM K/NaPO_4_, 1mM ethilendiaminotetraacetic acid /EDTA/, pH 7.0). Dorsolateral frontal cortex was isolated from 4.2 mm up to -1.32 mm from bregma [28]. After homogenization the crude synaptosomal fraction for determination of lipid peroxidation, the activity of superoxide dismutase (SOD), glutathione peroxidase (GPx), glutathione reductase (GR) and AchE, as well as reduced glutathione (GSH) level, was prepared according to the method of Whittaker and Barker [29]. Briefly, homogenates were centrifuged twice at 1000×g for 15 min at 4°C. The supernatants were further centrifuged at 20.000×g for 30 min. Supernatant obtained by this procedure represents crude synaptosomal fraction containing membrane vesicles (microsomes) from smooth and rough endoplasmic reticulum, Golgi and plasma membrane and all soluble components of the cytoplasm. Protein concentration was determined by the method of Lowry *et al* [30], using bovine albumin as a standard.

Lipid peroxidation, measured as malondialdehyde (MDA) level, was determined spectrophotometrically in a reaction with thiobarbituric acid. Thiobarbituric acid reacts with MDA released from polyunsaturated fatty acids forming a yellow complex whose absorbance is measured at 533 nm [31]. Results are expressed as nmol of MDA per milligram of proteins (nmol/mg protein).

Total SOD activity was assayed as the ability of crude synaptosomal fraction to inhibit the free radical-mediated autooxidation of epinephrine [32].

The content of reduced glutathione (GSH) was determined spectrophotometrically using 5,5-dithio-bis-2-nitrobenzoic acid (DTNB). DTNB reacts with aliphatic thiol compounds at pH 8.0 forming yellow p-nitrophenol anion whose absorption is measured spectrophotometrically at 412 nm [33]. Results are expressed as nmol per milligram of proteins (nmol/mg protein).

GPx activity was determined on the basis of oxidation of GSH with GPx using NADPH in a reaction catalyzed by enzyme GR. Decrease of absorbance at 340nm as a result of used NADPH+H^+^ represents the measure of GPx activity in coupled reaction with GR [34].

GR activity was assayed in a reaction of glutathione reduction in the presence of NADPH. NADPH consumption is proportional to the GR activity and was measured at 340 nm [35].

Catalase activity was determined in a reaction of H_2_O_2_ with ammonium-molibdate, which resulted in the formation of yellow complex, and its absorbance was measured at 405 nm [36].

AchE activity was measured spectrophotometrically at 412 nm, based on the formation of stable yellow complex in a reaction of DTNB with acetylcholine iodide degradation product [37].

All enzyme activities are expressed as units per milligram of protein (U/mg prot.).

### Western blot analysis

For Western blot analysis dorsolateral frontal cortex and hippocampus (n = 6 per group) were dissected and homogenized in lysis buffer (50 mMTris–HCl pH 7.4, 150 mMNaCl, 1% IGEPAL CA-630, 1 mM phenylmethylsulphonyl fluoride (Sigma-Aldrich, P7626), protease inhibitor cocktail (Sigma-Aldrich, P8340), 200 mM sodium orthovanadate (Sigma, Germany) and 1 M NaF (Merck, USA)) on ice for 30 min, followed by centrifugation (14.000 *g* for 15 min at 4°C), and the supernatants were collected as the cell lysates. Protein concentrations were determined by the method of Bradford [38] using bovine serum albumin as a standard (Sigma). Equal amounts of protein (50μg) from each sample were separated by sodium dodecylsulphate polyacrylamide gel electrophoresis (SDS-PAGE) on 10% and 12% gels and transferred to nitrocellulose membranes (Bio-Rad, Hercules, CA). Membranes were blocked at room temperature for 1 hour in 5% nonfat dry milk in Tris-buffered saline/0.1% Tween 20 (TBST). The following primary antibodies were used in this study: polyclonal goat anti-SOD1 (1:500, Santa Cruz, CA) and polyclonal goat anti-SOD2 (1:500, Santa Cruz, CA). After incubation with primary antibodies the membranes were incubated with the horseradish peroxidase (HRP) labeled secondary anti-goat antibody (1:2000, Southern Biotech, USA) in TBST for 1 hour at room temperature. Five subsequent washes with 0.1% TBST were performed between each step. All membranes were stripped and re-probed with anti-actin antibodies (1:10000, mouse monoclonal, Sigma, USA) to ensure that all wells were equally loaded. The signal was detected by enhanced chemiluminescence and subsequent exposure on an X-ray film. Western blots were scanned and densitometric analysis was performed using *ImageQuant 5*.*2*.

### Statistical analysis and drugs

All results are expressed as means±SD. The significance of the difference was estimated by using analysis of variance (ANOVA) with Fisher’s *post hoc* test, while the significance of the correlation between AchE activity and MDA level was determined by Pearson’s correlation test. The difference and correlation were considered significant if p<0.05. For statistical analysis computer software SPSS15.0 was used.

TAA, FIN and 2-hydroxypropyl-β-cyclodextrin were products of Sigma-Aldrich Chemical Co., U.S.A.

## Results

**Fig 1:**
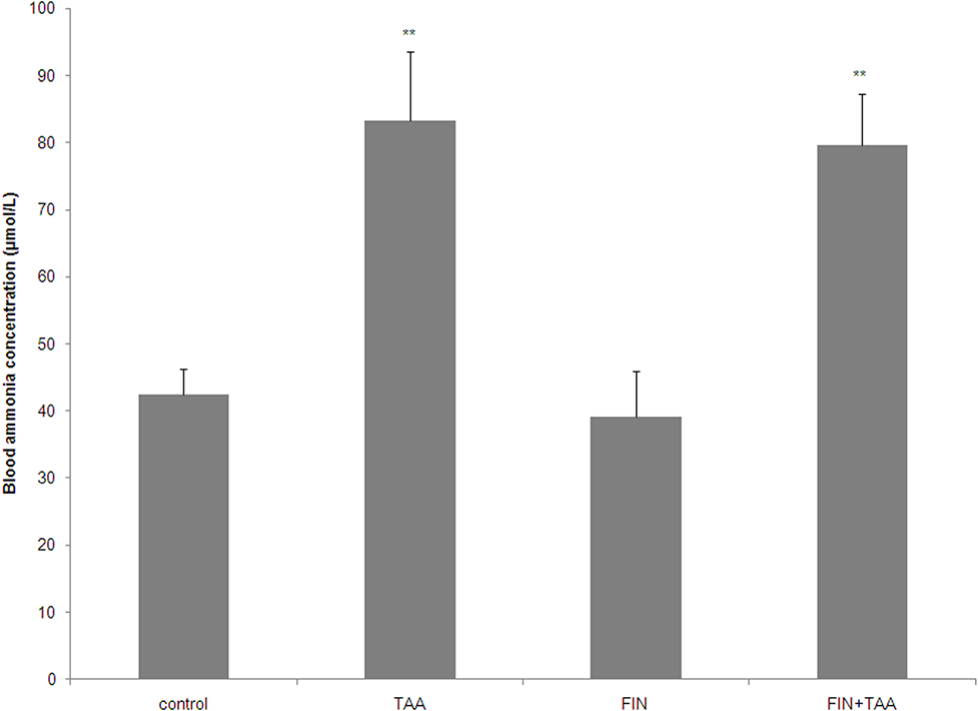
The effect of finasteride (FIN) and thioacetamide (TAA) on plasma ammonia concentration. Daily doses of FIN (50 mg/kg) and TAA (300 mg/kg) were administered intraperitoneally in three subsequent days and in FIN+TAA group FIN was administered 2 h before every dose of TAA. Blood samples were collected from the right side of the heart 24 h after administration of the last dose of TAA. The significance of the difference was estimated by ANOVA with Fisher’s *post hoc* test (**p<0.01 *vs*. control).

Blood ammonia concentration was significantly higher in TAA (83.2 ± 10.3 μmol/L, p<0.01) and FIN+TAA group (79.6 ± 7.6 μmol/L, p<0.01) by comparison with control (42.4 ± 3.9 μmol/L). No significant difference in ammonia concentration was evident between TAA and FIN+TAA group (p>0.05). FIN alone also did not cause any change in ammonia concentration (Fig 1).

**Fig 2:**
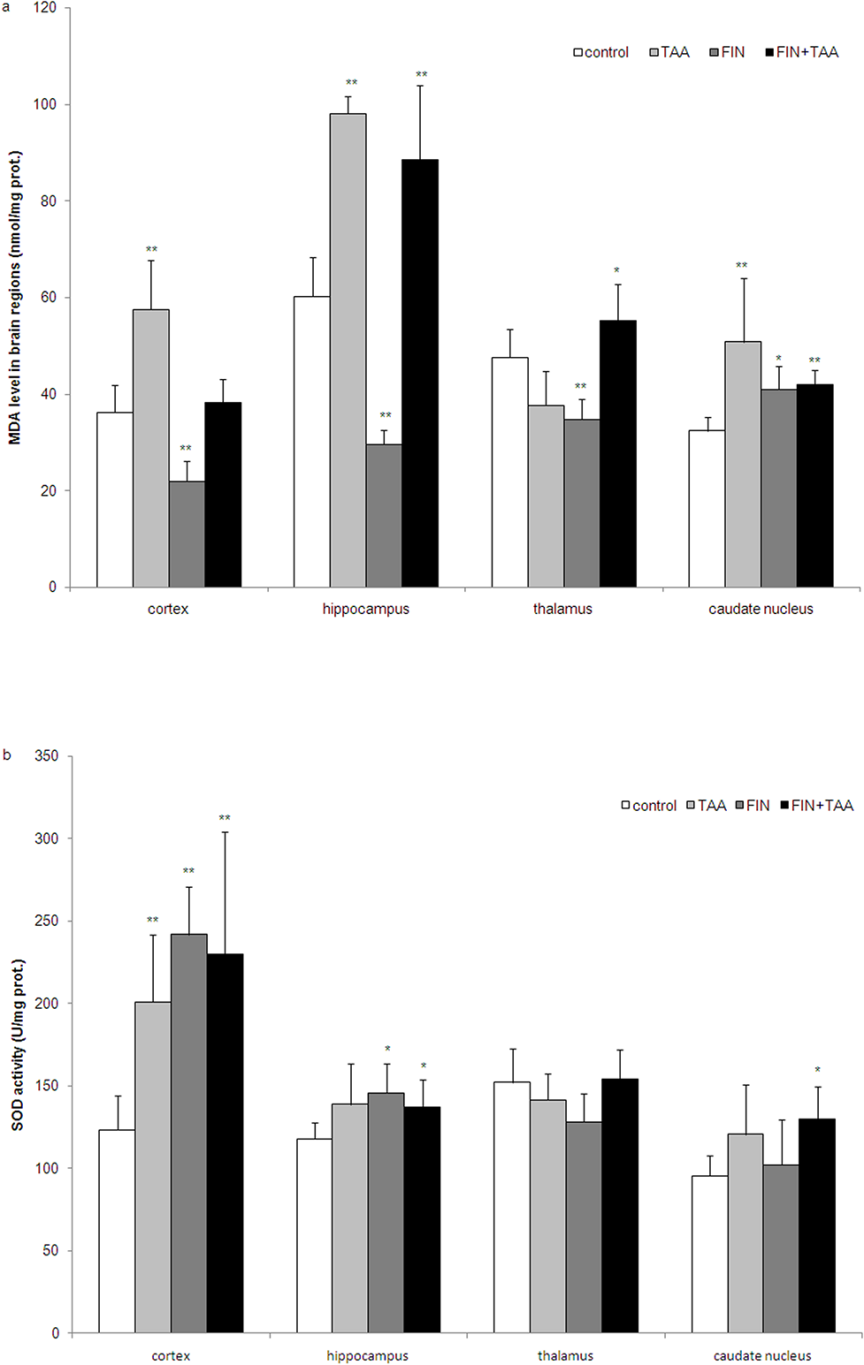
The effects of finasteride (FIN) and thioacetamide (TAA) on (a) malondialdehyde (MDA) level and (b) superoxide dismutase (SOD) activity in various brain regions. Four brain regions (dorsolateral frontal cortex, hippocampus, thalamus and caudate nucleus) were isolated 24 h after administration of the last dose of TAA and crude synaptosomal fractions were prepared for determination of parameters of oxidative stress. The significance of the difference was estimated by ANOVA with Fisher’s *post hoc* test (*p<0.05 and **p<0.01 *vs*. control in the same region). For details see caption for Fig 1.

MDA level was significantly higher in the cortex, the hippocampus and caudate nucleus in TAA *vs*. control group 24 h after the administration of the last dose of TAA (p<0.01). In contrast, TAA did not induce a significant change in MDA level in the thalamus (37.68 ± 7.13 nmol/mg prot.) when compared with control (47.62 ± 5.81 nmol/mg prot., p>0.05). While FIN alone reduced MDA level in the cortex (21.99 ± 4.02 nmol/mg prot., p<0.01), the hippocampus (29.53 ± 2.96 nmol/mg prot., p<0.01) and the thalamus (34.82 ± 4.14 nmol/mg prot., p<0.01), and increased its level in caudate nucleus (40.93 ± 4.92 nmol/mg prot., p<0.05), in FIN+TAA group MDA level was significantly higher in hippocampus (88.63 ± 15.24 nmol/mg prot., p<0.01), caudate nucleus (42.07 ± 2.79 nmol/mg prot., p<0.01) and thalamus when compared with control (60.20 ± 8.13 and 32.40 ± 2.86 nmol/mg prot. in hippocampus and caudate nucleus respectively, Fig 2A).

SOD activity was significantly higher only in the cortex of animals from TAA group (201.02 ± 40.47 U/mg prot.) when compared with control (123.32 ± 20.75 U/mg prot., p<0.01). However, in FIN+TAA group the activity of this enzyme was significantly higher in the cortex (229.79 ± 74.41 U/mg prot., p<0.01), the hippocampus (137.06 ± 17.02 U/mg prot., p<0.05) and caudate nucleus (129.57 ± 20.13 U/mg prot., p<0.05) by comparison with control. Only in thalamus no significant change was found between FIN+TAA (154.32 ± 17.85 U/mg prot.) and control group (152.11 ± 20.37 U/mg prot., p>0.05). Despite these changes, SOD activity was not significantly different in any brain region between FIN+TAA and TAA group (p>0.05). FIN alone induced a significant increase in SOD activity in the cortex (241.65 ± 28.97 U/mg prot., p<0.01) and the hippocampus (145.68 ± 18.04 U/mg prot., p<0.05) when compared with control (Fig 2B).

**Fig 3:**
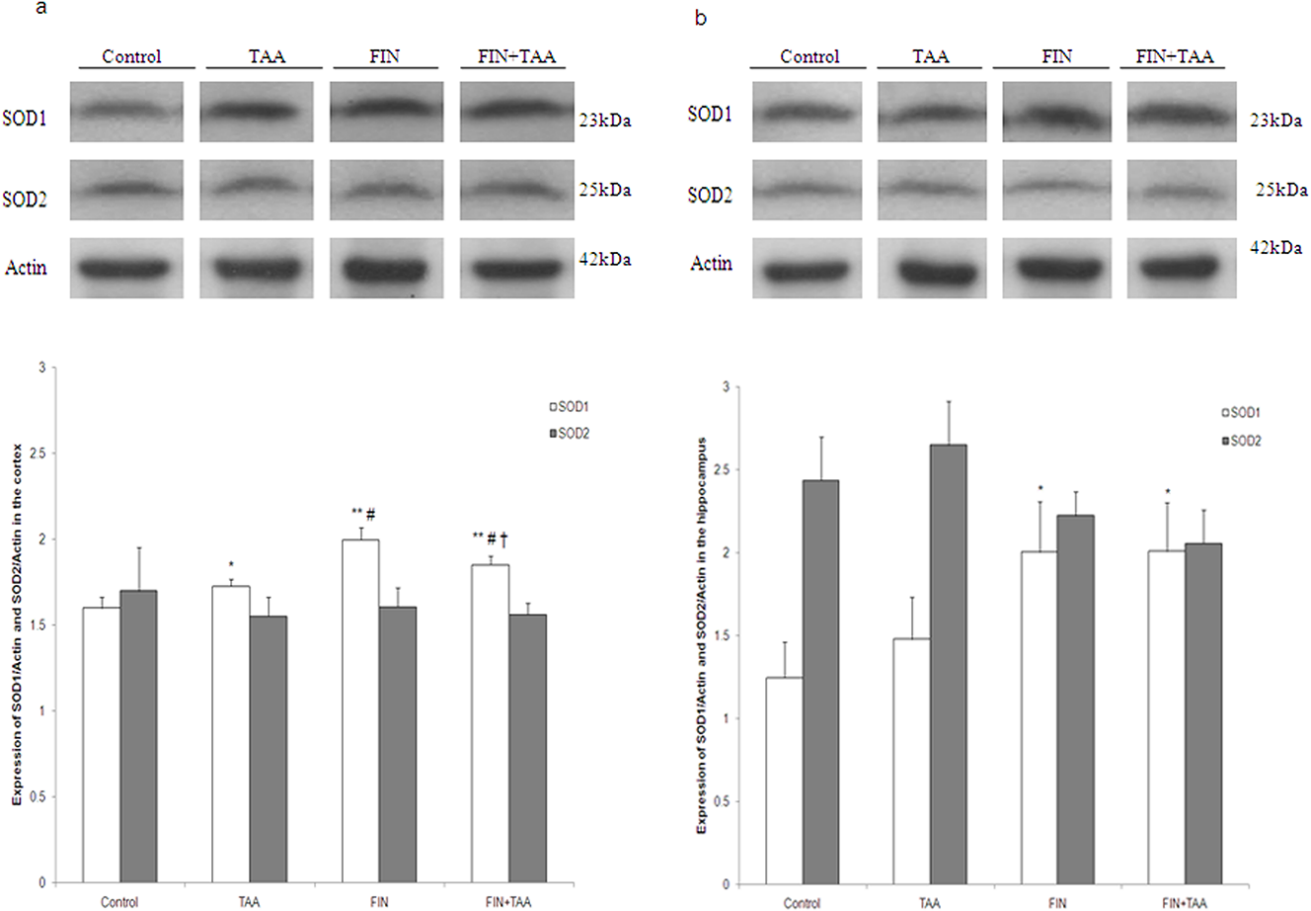
The effects of finasteride (FIN) and thioacetamide (TAA) on expression of cytosolic (SOD1) and mitochondrial SOD izoenzyme (SOD2) in the (a) cortex and (b) the hippocampus. For determination of expression polyclonal goat anti-SOD1 (1:500) and polyclonal goat anti-SOD2 (1:500) were used. After incubation with primary antibodies the membranes were incubated with the horseradish peroxidase (HRP) labeled secondary anti-goat antibody (1:2000) in TBST for 1 hour at room temperature. Five subsequent washes with 0.1% TBST were performed between each step. All membranes were stripped and re-probed with mouse monoclonal anti-actin antibodies (1:10000) to ensure that all wells were equally loaded. The signal was detected by enhanced chemiluminescence and subsequent exposure on an X-ray film. The significance of the difference was estimated by ANOVA with Fisher’s *post hoc* test (*p<0.05 and **p<0.01 *vs*. control, ##p<0.01 *vs*. TAA group, †p<0.01 *vs*. FIN group). For details see caption for Fig 1.

Analysis of SOD izoenzymes revealed that SOD1 expression in the cortex was significantly higher in all experimental groups *vs*. control. However, in FIN+TAA group the expression of this izoenzyme was significantly higher by comparison with TAA (p<0.01) and significantly lower when compared with FIN group (p<0.01, Fig 3A). In hippocampus SOD1 expression was significantly higher in FIN (p<0.05) and FIN+TAA group (p<0.05) by comparison with control, but no change in the expression of this izoenzyme in the hippocampus was evident in TAA *vs*. control group (p>0.05, Fig 3B). No significant changes in SOD2 expression among groups were evident in both brain regions.

**Fig 4:**
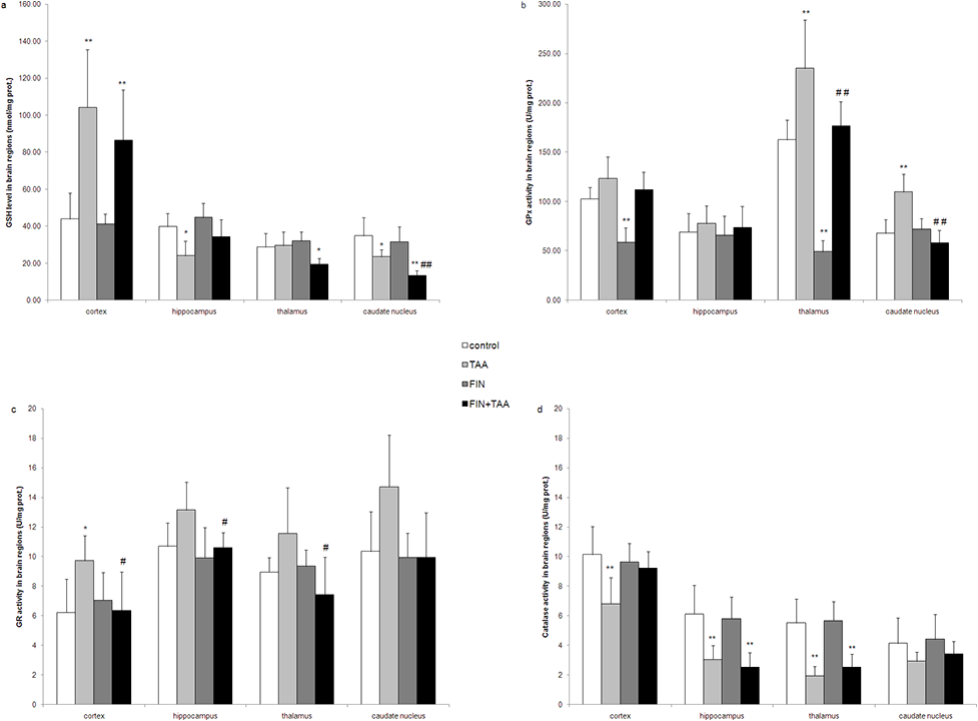
The effects of finasteride (FIN) and thioacetamide (TAA) on (a) reduced glutathione (GSH) level, (b) glutathione peroxidase (GPx), (c) glutathione reductase (GR) and (d) catalase activity in various brain regions. The significance of the difference was estimated by ANOVA with Fisher’s *post hoc* test (*p<0.05 and **p<0.01 *vs*. control, #p<0.05 and ##p<0.01 *vs*. TAA group in the same region). For details see caption for Figs 1 and 2.

GSH level in TAA group was significantly higher in the cortex (104.29 ± 31.24 nmol/mg prot., p<0.01) and significantly lower in the hippocampus (24.05 ± 7.92 nmol/mg prot., p<0.05) and caudate nucleus (23.46 ± 3.71 nmol/mg prot., p<0.05) when compared with control. Similar to TAA group, in FIN+TAA group GSH level was significantly higher in the cortex (86.52 ± 27.23 nmol/mg prot., p<0.01) and significantly lower in caudate nucleus (13.18 ± 2.79 nmol/mg prot., p<0.01) by comparison with control group (43.98 ± 14.04 nmol/mg prot. and 34.70 ± 9.91 nmol/mg prot. in the cortex and caudate nucleus respectively). However, in caudate nucleus GSH level was significantly lower in FIN+TAA *vs*. TAA group (p<0.01), while in the cortex no significant difference in GSH level was evident between these experimental groups (p>0.05). Hippocampal GSH level was not significantly different in FIN+TAA (34.26 ± 9.17 nmol/mg prot.) *vs*. control group (39.67 ± 7.11 nmol/mg prot., p>0.05). Additionally, while TAA alone did not induce significant changes in thalamic GSH level (p>0.05), its level in the thalamus was significantly lower in FIN+TAA group (19.37 ± 3.31 nmol/mg prot., p<0.05) by comparison with control (28.76 ± 7.37 nmol/mg prot., Fig 4A).

GPx activity was significantly higher in the thalamus (235.09 ± 49.12 U/mg prot., p<0.01) and caudate nucleus (109.61 ± 18.03 U/mg prot., p<0.01) of animals from TAA group by comparison with control (162.61 ± 20.11 U/mg prot. and 67.85 ± 13.60 U/mg prot. in the thalamus and caudate nucleus respectively). Although GPx activity was not different between FIN+TAA and control group in any brain region (p>0.05), the activity of this enzyme was significantly lower in the thalamus (176.71 ± 24.61 U/mg prot., p<0.01) and caudate nucleus (57.82 ± 13.20 U/mg prot., p<0.01) of animals from FIN+TAA when compared with TAA group. On the other hand FIN alone induced a decline in GPx activity in the cortex (58.38 ± 15.07 U/mg prot., p<0.01) and the thalamus (49.17 ± 11.37 U/mg prot., p<0.01) by comparison with control (Fig 4B).

GR activity was significantly higher only in the cortex of animals from TAA group (9.74 ± 1.67 U/mg prot., p<0.05) compared with control (6.23 ±2.24 U/mg prot.). No difference in the activity of this enzyme was evident between FIN, FIN+TAA and control group (p>0.05) in any brain region. However, GR activity in FIN+TAA group was significantly lower in the cortex (6.37 ± 2.61 U/mg prot., p<0.05), the thalamus (7.44 ± 2.53 U/mg prot., p<0.05) and the hippocampus (10.61 ± 1.01 U/mg prot., p<0.05) by comparison with TAA group (Fig 4C).

Catalase activity in TAA group was significantly lower in the cortex (6.81 ± 1.77 U/mg prot., p<0.01), the hippocampus (3.06 ± 0.92 U/mg prot., p<0.01) and the thalamus (1.96 ± 0.61 U/mg prot., p<0.01) when compared with control. Although FIN alone did not induce significant changes in catalase activity, in FIN+TAA group the activity of this enzyme was significantly lower in the hippocampus (2.54 ± 0.98 U/mg prot., p<0.01) and the thalamus (2.52 ± 0.88 U/mg prot., p<0.01) compared with control (6.13 ± 1.93 U/mg prot. and 5.53 ± 1.60 U/mg prot. in the hippocampus and the thalamus respectively, Fig 4D).

**Fig 5:**
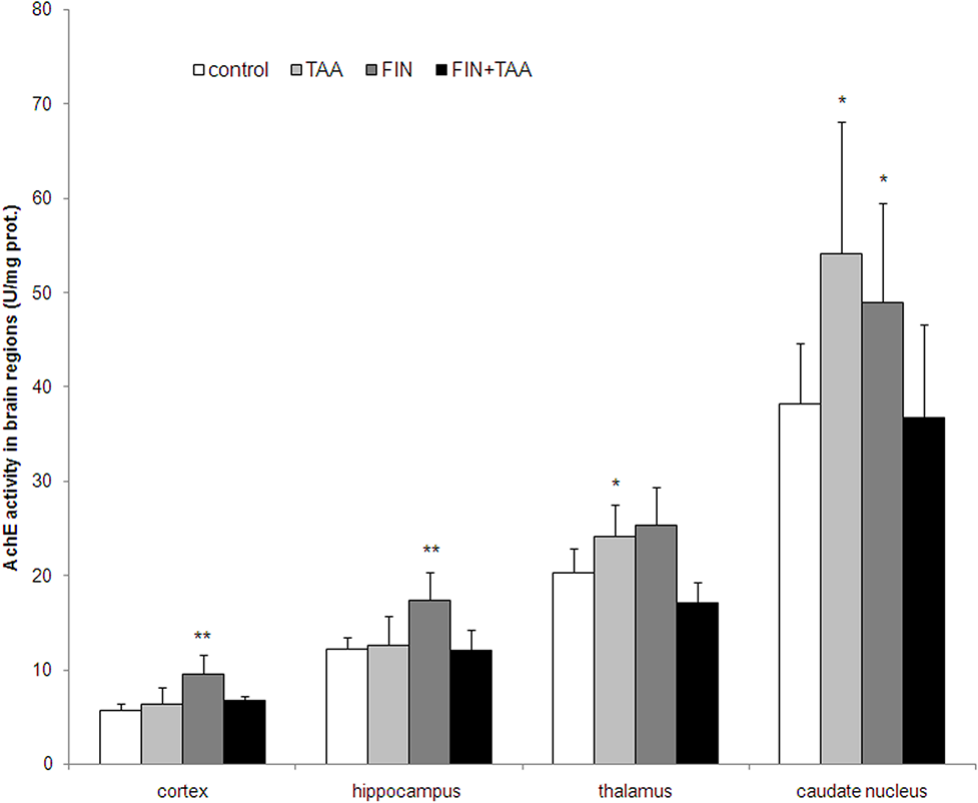
The effects of finasteride (FIN) and thioacetamide (TAA) on acetylcholinesterase (AchE) activity in various brain regions. The significance of the difference was estimated by ANOVA with Fisher’s *post hoc* test (*p<0.05 and **p<0.01 *vs*. control in the same region). For details see caption for Figs 1 and 2.

AchE activity was significantly higher in the thalamus (24.16 ± 3.37 U/mg prot., p<0.05) and caudate nucleus of animals from TAA (54.10 ± 14.06 U/mg prot., p<0.05) *vs*. control group (20.31 ± 2.58 U/mg prot. and 38.20 ± 6.39 U/mg prot. in the thalamus and caudate nucleus respectively). Although FIN alone caused an increase in AchE activity in the cortex (9.53 ± 1.99 U/mg prot., p<0.01), the hippocampus (17.44 ± 2.82 U/mg prot., p<0.01) and caudate nucleus (48.98 ± 10.45 U/mg prot., p<0.05) when compared with control, the activity of this enzyme was not different between FIN+TAA and control group in any brain region (p>0.05, Fig 5).

**Fig 6:**
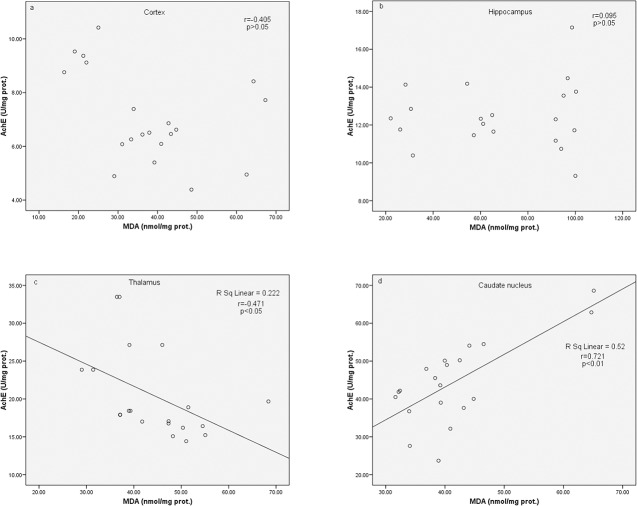
The correlation between acetylcholinesterase (AchE) activity and malondialdehyde (MDA) level in various brain regions. The significance of the correlation was estimated by Pearson’s correlation test. For details see caption for Figs 1 and 2.

AchE activity was found to correlate negatively with MDA level in the thalamus (p<0.05) and positively with MDA level in caudate nucleus (p<0.01). No correlation between AchE activity and MDA level was found in the cortex and the hippocampus (p>0.05, Fig 6).

## Discussion

Through reduction of lipid peroxidation and nitrozative stress FIN has beneficial effects in the treatment of prostatic hyperplasia [39]. In contrast to the prostate, previous studies [40,41] have shown possible dual effects of FIN on oxidative stress in the brain. While this drug was found to attenuate palmitoilethanolamide-induced lipid peroxidation in cultured glioma cells [40], oxidative brain injury in orofacial dyskinesia was aggravated after FIN treatment [41].

Our previous study [27] as well as blood ammonia rise caused by TAA (Fig 1) clearly show that TAA in a dose of 900 mg/kg is suitable for induction of severe HE, including hepatic coma in rats. This is the first study that investigated the effects of FIN on oxidative stress in the brain in HE.

The present study indicates that FIN has different roles in the modulation of oxidative stress in acute HE in various brain regions. TAA-induced lipid peroxidation was alleviated in the cortex and aggravated in the thalamus after FIN pretreatment (Fig 2A). Regional differences in the effects of FIN on oxidative stress in the brain are not related to hyperammonemia, since FIN did not prevent TAA-induced rise in blood ammonia (Fig 1), but may be explained by several other mechanisms.

First, regional differences may be caused by different effects of FIN on antioxidative enzymes in various brain regions. Aggravation of lipid peroxidation by FIN in the thalamus in acute TAA-induced HE was associated with decline of GSH level and may be explained by reduction in GR activity and partly by suppression of TAA-induced increase in GPx activity (Fig 4A–4C). GPx potentially has a significant role in the defense of thalamus from ROS, since the activity of this enzyme under basal conditions was the highest in this region (Fig 4B). However, since FIN alone reduced lipid peroxidation in the thalamus along with decline in GPx activity (Figs 2A and 4B), the prooxidative role of FIN in the thalamus in TAA-induced HE is caused by some additional mechanisms.

On the other hand, reduction of lipid peroxidation in the cortex after FIN pretreatment was associated with restoration of catalase activity (Figs 2A and 4D). Previous studies [42,43] along with the present study clearly indicate that the activity of antioxidative enzymes in the cortex is influenced by the stage of HE. While catalase has an important role in antioxidant defense in mild HE [42], moderate HE is accompanied with an increase in mitochondrial izoenzyme SOD2 [43]. According to our study, SOD1 and GR seem to have a crucial role in adaptation to oxidative injury in severe HE (Figs 3A and 4C). This indicates that the effects of FIN on lipid peroxidation in the cortex may depend on the stage of HE and one of mechanisms involved in the antioxidant effects of FIN in severe TAA-induced HE may be prevention of decrease in catalase activity (Fig 4D), the enzyme that removes ROS in combination with SOD. Interestingly, FIN pretreatment prevented TAA-induced increase in GR (Fig 4C), but not increase in cortical GSH level (Fig 4A). The possible explanation for this finding may be increased H_2_O_2_ neutralization by catalase with subsequent decreased production of more potent ROS that would alter the redox state of the cells.

Although FIN had no significant effects on TAA-induced lipid peroxidation in the hippocampus and caudate nucleus, this drug caused changes in the activities of antioxidative enzymes (Figs 2 and 4). Similar to the cortex, the expression of SOD1 was increased by FIN pretreatment also in the hippocampus in TAA-induced HE (Fig 3B), which indicates that FIN stimulates dismutation as a possible adaptive response to TAA-induced lipid peroxidation. However, the hippocampus appears to be even more susceptible to oxidative injury after FIN pretreatment because of a reduction in GR activity (Fig 4C). This indicates that FIN may have potentially beneficial effects on oxidative stress in the hippocampus in combination with other antioxidants, such as curcumin. Curcumin was found to increase hippocampal activity of SOD, GR and GPx in rats in HE induced by portal vein ligation [44]. Despite reduction in GR activity, a decline in hippocampal GSH level induced by TAA was prevented by FIN (Fig 4A). The complexity of FIN effects on hippocampal oxidative status may be related to the different vulnerability of various hippocampal regions to oxidative stress. In vitro studies have found that CA1 region is vulnerable, while CA3 region is resistant to oxidative stress, evident as different neuronal gene expression patterns in these regions [45].

Redox state estimated according to GSH level was found to be shifted towards oxidants in caudate nucleus after FIN pretreatment (Fig 4A). Although FIN pretreatment caused an increase in SOD activity (Fig 2B), a decline in GSH level indicates that donors of sulfhydryl groups may have more beneficial effects in prevention of oxidative injury in caudate nucleus in HE than FIN. Oxidative injury of caudate nucleus may contribute to the motor disturbances in HE [46].

The second potential explanation for regional differences of FIN effects on oxidative stress in the brain in TAA-induced HE may be related to the effects of FIN on AchE activity. Although evident, the role of AchE in the pathogenesis of HE is still not completely understood. Studies in patients with liver cirrhosis have found an increase in AchE activity in the brain [13], while in TAA-induced model of cirrhosis the activity of AchE was found to be elevated in enthorinal cortex, nc. accumbens, anterodorsal and anteroventral thalamus, and decreased in CA1, CA3 region and dentate gyrus of hippocampus [47]. On the other hand, Zarros et al. [48] have not found changes in AchE activity in acute HE, while Swapna et al. [49], in contrast to our study, have observed a decline in this enzyme activity in the cortex after acute TAA administration. These discrepancies may be explained by different doses of TAA used in these studies and partly by different mechanisms of HE development in various models. The complexity of AchE effects on the pathogenesis of HE may be further confirmed by improvement of cognitive, but not motor functions, after AchE inhibition by rivastigmin in rat liver failure [13].

However, the link between oxidative stress in the brain and AchE activity is less controversial. Long-term inhibition of AchE was found to increase the activity of various brain regions with subsequent adenosine triphosphate (ATP) and creatine phosphate depletion in the neurons. Additionally, AchE inhibition impairs oxidative phosphorylation and is followed by neuronal Ca^2+^ influx and activation of nNOS, associated with oxidative and nitrozative injury of the neurons [50]. This is the first study that suggests that FIN has regionally selective modulatory effect on cholinergic transmission and that inhibition of AchE may be at least partly responsible for adverse effects of FIN treatment on lipid peroxidation in the thalamus, but not in other brain regions in acute TAA-induced HE (Figs 5 and 6).

Interestingly, lipid peroxidation was found to correlate positively with AchE activity in caudate nucleus (Fig 6D). This possibly indicates that opposite to thalamus, further AchE inhibition may even reduce lipid peroxidation in caudate nucleus, and that modulation of cholinergic transmission in these regions may have opposite effects on oxidative stress and neuronal function. The mechanisms of different effects of FIN on AchE and its correlation with oxidative stress in various brain regions should be further investigated, but one of potential mechanisms may be related to regional differences in AchE activity under basal conditions. AchE activity is the highest in diencephalon (thalamus and hypothalamus) and the lowest in the cerebellum and neocortex [51]. Despite these differences, our findings imply that modulation of cholinergic transmission may be an additional therapeutical strategy in HE apart from detoxication of ammonia, reduction in gut flora and modulation of GABAergic transmission [52].

Finally, the last potential mechanism of regional selectivity of FIN effects on oxidative stress in the brain may be alterations in metabolism induced by FIN. In the prostate FIN has been found to decrease the expression of α chain of ATP synthase, farnesyl diphosphate synthase and phosphofructokinase 2/fructose-2,6-bisphosphatase and to increase the expression of transketolase, aldolase, glyceraldehyde-3-phosphate dehydrogenase and pyruvate kinase [53]. The effects of FIN on the activity of these enzymes in the brain and their link with oxidative stress has to be further investigated.

Based on our results, it can be thrash out that FIN has regional and selective effects on oxidative stress and AchE activity in the brain in acute TAA-induced HE in rats.

FIN reduces lipid peroxidation in the cortex due to increase in catalase activity and at the same time increases expression of cytosolic SOD1. In contrast, FIN aggravates lipid peroxidation in the thalamus due to reduction in GR and suppression of TAA-induced rise in GPx activity. The prooxidant role of FIN may be causally linked with inhibition of AchE in the thalamus. AchE activity correlates with the degree of lipid peroxidation in caudate nucleus. Although FIN induces changes in antioxidative enzymes in the hippocampus and the cortex, it has no effect on lipid peroxidation in these brain regions in acute TAA-induced HE in rats.

## Supporting Information

S1 Dataset Raw data used in the present study. (DOCX)
