# Supplementary material for: Finasteride Has Regionally Different Effects on Brain Oxidative Stress and Acetylcholinesterase Activity in Acute Thioacetamide-Induced Hepatic Encephalopathy in Rats
Source: PLoS One. 2015 Aug 4;10(8):e0134434. doi: 10.1371/journal.pone.0134434 (PMC4524603; doi:10.1371/journal.pone.0134434)
Supplement: S1 Dataset — (DOCX) [file pone.0134434.s001.docx]

|  | ammonia | |  |  | |  | |  | | |  | |
| --- | --- | --- | --- | --- | --- | --- | --- | --- | --- | --- | --- | --- |
|  | control |  | TAA |  | | FIN | |  | | | FIN+TAA | |
| C1 | 42.6 | TAA1 | 88.2 | FIN1 | | 49.6 | | FIN+TAA1 | | | 75.9 | |
| C2 | 37.1 | TAA2 | 77.7 | FIN2 | | 35.2 | | FIN+TAA2 | | | 76.7 | |
| C3 | 40.5 | TAA3 | 83.2 | FIN3 | | 41.6 | | FIN+TAA3 | | | 73.3 | |
| C4 | 44.3 | TAA4 | 87.0 | FIN4 | | 42.1 | | FIN+TAA4 | | | 74.1 | |
| C5 | 39.7 | TAA5 | 96.9 | FIN5 | | 32.4 | | FIN+TAA5 | | | 76.5 | |
| C6 | 43.0 | TAA6 | 86.8 | FIN6 | | 33.5 | | FIN+TAA6 | | | 88.6 | |
| C7 | 41.3 | TAA7 | 95.6 | FIN7 | | 45.6 | | FIN+TAA7 | | | 94.2 | |
| C8 | 50.3 | TAA8 | 78.2 | FIN8 | | 32.8 | | FIN+TAA8 | | | 77.3 | |
| mean | 42.4 | TAA9 | 81.7 | mean | | 39.1 | | mean | | | 79.6 | |
| SD | 3.9 | TAA10 | 92.2 | sd | | 6.6 | | sd | | | 7.6 | |
|  |  | TAA11 | 88.1 |  | |  | |  | | |  | |
|  |  | TAA12 | 89.9 |  | |  | |  | | |  | |
|  |  | TAA13 | 96.7 |  | |  | |  | | |  | |
|  |  | TAA14 | 72.6 |  | |  | |  | | |  | |
|  |  | TAA15 | 66.8 |  | |  | |  | | |  | |
|  |  | TAA16 | 69.1 |  | |  | |  | | |  | |
|  |  | TAA17 | 84.4 |  | |  | |  | | |  | |
|  |  | TAA18 | 62.8 |  | |  | |  | | |  | |
|  |  | mean | 83.2 |  | |  | |  | | |  | |
|  |  | SD | 10.3 |  | |  | |  | | |  | |
|  | MDA cort |  |  |  | |  | |  | | |  | |
|  | control |  | TAA |  | | FIN | |  | | | FIN+TAA | |
| C1 | 35.20 | TAA1 | 56.61 | FIN1 | | 27.58 | | FIN+TAA1 | | | 31.51 | |
| C2 | 30.85 | TAA2 | 61.65 | FIN2 | | 23.64 | | FIN+TAA2 | | | 35.36 | |
| C3 | 42.56 | TAA3 | 61.83 | FIN3 | | 21.42 | | FIN+TAA3 | | | 33.45 | |
| C4 | 31.06 | TAA4 | 62.57 | FIN4 | | 17.20 | | FIN+TAA4 | | | 35.98 | |
| C5 | 30.65 | TAA5 | 62.90 | FIN5 | | 15.80 | | FIN+TAA5 | | | 40.48 | |
| C6 | 33.05 | TAA6 | 75.22 | FIN6 | | 24.99 | | FIN+TAA6 | | | 43.17 | |
| C7 | 43.85 | TAA7 | 54.24 | FIN7 | | 24.62 | | FIN+TAA7 | | | 42.42 | |
| C8 | 42.18 | TAA8 | 42.63 | FIN8 | | 20.66 | | FIN+TAA8 | | | 43.79 | |
| mean | 36.17 | TAA9 | 49.89 | mean | | 21.99 | | mean | | | 38.27 | |
| SD | 5.75 | TAA10 | 45.98 | sd | | 4.02 | | sd | | | 4.77 | |
|  |  | TAA11 | 42.73 |  | |  | |  | | |  | |
|  |  | TAA12 | 64.53 |  | |  | |  | | |  | |
|  |  | TAA13 | 68.64 |  | |  | |  | | |  | |
|  |  | TAA14 | 58.23 |  | |  | |  | | |  | |
|  |  | TAA15 | 51.41 |  | |  | |  | | |  | |
|  |  | TAA16 | 50.33 |  | |  | |  | | |  | |
|  |  | TAA17 | 48.85 |  | |  | |  | | |  | |
|  |  | TAA18 | 76.58 |  | |  | |  | | |  | |
|  |  | mean | 57.49 |  | |  | |  | | |  | |
|  |  | SD | 10.14 |  | |  | |  | | |  | |
|  | MDA hipp | |  | |  | |  | |  |  | |  |
|  | control |  | TAA | |  | | FIN | |  | FIN+TAA | |  |
| C1 | 69.50 | TAA1 | 91.19 | | FIN1 | | 27.08 | | FIN+TAA1 | 110.39 | |  |
| C2 | 67.72 | TAA2 | 98.34 | | FIN2 | | 26.25 | | FIN+TAA2 | 80.42 | |  |
| C3 | 53.80 | TAA3 | 101.21 | | FIN3 | | 32.31 | | FIN+TAA3 | 101.92 | |  |
| C4 | 48.28 | TAA4 | 100.03 | | FIN4 | | 28.84 | | FIN+TAA4 | 83.37 | |  |
| C5 | 56.63 | TAA5 | 92.76 | | FIN5 | | 26.32 | | FIN+TAA5 | 73.91 | |  |
| C6 | 63.65 | TAA6 | 91.42 | | FIN6 | | 30.97 | | FIN+TAA6 | 71.92 | |  |
| C7 | 53.60 | TAA7 | 100.07 | | FIN7 | | 30.07 | | FIN+TAA7 | 80.60 | |  |
| C8 | 68.45 | TAA8 | 101.55 | | FIN8 | | 34.38 | | FIN+TAA8 | 106.51 | |  |
| mean | **60.20** | TAA9 | 96.45 | | mean | | **29.53** | | mean | **88.63** | |  |
| SD | **8.13** | TAA10 | 100.95 | | sd | | **2.96** | | sd | **15.24** | |  |
|  |  | TAA11 | 99.97 | |  | |  | |  |  | |  |
|  |  | TAA12 | 100.15 | |  | |  | |  |  | |  |
|  |  | TAA13 | 93.57 | |  | |  | |  |  | |  |
|  |  | TAA14 | 95.53 | |  | |  | |  |  | |  |
|  |  | TAA15 | 100.05 | |  | |  | |  |  | |  |
|  |  | TAA16 | 100.77 | |  | |  | |  |  | |  |
|  |  | TAA17 | 102.24 | |  | |  | |  |  | |  |
|  |  | TAA18 | 98.56 | |  | |  | |  |  | |  |
|  |  | mean | **98.05** | |  | |  | |  |  | |  |
|  |  | SD | **3.63** | |  | |  | |  |  | |  |
|  | MDA thal |  |  | |  | |  | |  |  | |  |
|  | control |  | TAA | |  | | FIN | |  | FIN+TAA | |  |
| C1 | 44.33 | TAA1 | 34.83 | | FIN1 | | 39.15 | | FIN+TAA1 | 48.07 | |  |
| C2 | 46.00 | TAA2 | 39.25 | | FIN2 | | 26.42 | | FIN+TAA2 | 48.92 | |  |
| C3 | 54.01 | TAA3 | 33.57 | | FIN3 | | 33.19 | | FIN+TAA3 | 45.14 | |  |
| C4 | 52.55 | TAA4 | 37.23 | | FIN4 | | 34.45 | | FIN+TAA4 | 60.37 | |  |
| C5 | 50.27 | TAA5 | 45.75 | | FIN5 | | 35.84 | | FIN+TAA5 | 58.08 | |  |
| C6 | 53.68 | TAA6 | 47.54 | | FIN6 | | 35.65 | | FIN+TAA6 | 64.88 | |  |
| C7 | 40.52 | TAA7 | 50.98 | | FIN7 | | 39.85 | | FIN+TAA7 | 52.31 | |  |
| C8 | 39.64 | TAA8 | 36.64 | | FIN8 | | 33.98 | | FIN+TAA8 | 63.49 | |  |
| mean | 47.62 | TAA9 | 35.79 | | mean | | 34.82 | | mean | 55.16 | |  |
| SD | 5.81 | TAA10 | 43.19 | | sd | | 4.14 | | sd | 7.53 | |  |
|  |  | TAA11 | 40.05 | |  | |  | |  |  | |  |
|  |  | TAA12 | 34.09 | |  | |  | |  |  | |  |
|  |  | TAA13 | 33.16 | |  | |  | |  |  | |  |
|  |  | TAA14 | 49.13 | |  | |  | |  |  | |  |
|  |  | TAA15 | 28.62 | |  | |  | |  |  | |  |
|  |  | TAA16 | 27.62 | |  | |  | |  |  | |  |
|  |  | TAA17 | 31.70 | |  | |  | |  |  | |  |
|  |  | TAA18 | 29.18 | |  | |  | |  |  | |  |
|  |  | mean | **37.68** | |  | |  | |  |  | |  |
|  |  | SD | **7.13** | |  | |  | |  |  | |  |
|  | MDA nc |  |  | |  | |  | |  |  | |  |
|  | control |  | TAA | |  | | FIN | |  | FIN+TAA | |  |
| C1 | 34.62 | TAA1 | 70.92 | | FIN1 | | 36.24 | | FIN+TAA1 | 44.92 | |  |
| C2 | 31.66 | TAA2 | 34.39 | | FIN2 | | 47.93 | | FIN+TAA2 | 42.37 | |  |
| C3 | 27.22 | TAA3 | 56.55 | | FIN3 | | 41.82 | | FIN+TAA3 | 47.10 | |  |
| C4 | 30.83 | TAA4 | 30.18 | | FIN4 | | 36.95 | | FIN+TAA4 | 39.38 | |  |
| C5 | 35.75 | TAA5 | 53.90 | | FIN5 | | 37.16 | | FIN+TAA5 | 41.96 | |  |
| C6 | 32.25 | TAA6 | 36.21 | | FIN6 | | 46.96 | | FIN+TAA6 | 40.80 | |  |
| C7 | 31.29 | TAA7 | 51.94 | | FIN7 | | 36.32 | | FIN+TAA7 | 41.29 | |  |
| C8 | 35.54 | TAA8 | 31.14 | | FIN8 | | 44.05 | | FIN+TAA8 | 38.70 | |  |
| mean | 32.40 | TAA9 | 32.01 | | mean | | 40.93 | | mean | 42.07 | |  |
| SD | 2.86 | TAA10 | 41.36 | | sd | | 4.92 | | sd | 2.79 | |  |
|  |  | TAA11 | 61.39 | |  | |  | |  |  | |  |
|  |  | TAA12 | 56.69 | |  | |  | |  |  | |  |
|  |  | TAA13 | 69.58 | |  | |  | |  |  | |  |
|  |  | TAA14 | 56.50 | |  | |  | |  |  | |  |
|  |  | TAA15 | 57.10 | |  | |  | |  |  | |  |
|  |  | TAA16 | 60.00 | |  | |  | |  |  | |  |
|  |  | TAA17 | 51.97 | |  | |  | |  |  | |  |
|  |  | TAA18 | 62.59 | |  | |  | |  |  | |  |
|  |  | mean | **50.80** | |  | |  | |  |  | |  |
|  |  | SD | **13.24** | |  | |  | |  |  | |  |

|  | SOD cort |  |  |  |  |  |  |
| --- | --- | --- | --- | --- | --- | --- | --- |
|  | control |  | TAA |  | FIN |  | FIN+TAA |
| C1 | 140.33 | TAA1 | 176.68 | FIN1 | 230.72 | FIN+TAA1 | 328.06 |
| C2 | 124.58 | TAA2 | 242.86 | FIN2 | 231.49 | FIN+TAA2 | 128.29 |
| C3 | 117.31 | TAA3 | 181.97 | FIN3 | 210.11 | FIN+TAA3 | 162.91 |
| C4 | 139.53 | TAA4 | 240.58 | FIN4 | 220.50 | FIN+TAA4 | 209.95 |
| C5 | 149.23 | TAA5 | 164.38 | FIN5 | 278.56 | FIN+TAA5 | 275.57 |
| C6 | 101.57 | TAA6 | 257.51 | FIN6 | 272.07 | FIN+TAA6 | 196.45 |
| C7 | 126.31 | TAA7 | 231.84 | FIN7 | 275.71 | FIN+TAA7 | 206.43 |
| C8 | 87.69 | TAA8 | 243.89 | FIN8 | 214.03 | FIN+TAA8 | 330.66 |
| mean | **123.32** | TAA9 | 223.23 | mean | **241.65** | mean | **229.79** |
| SD | **20.75** | TAA10 | 174.78 | sd | **28.97** | sd | **74.41** |
|  |  | TAA11 | 159.29 |  |  |  |  |
|  |  | TAA12 | 229.49 |  |  |  |  |
|  |  | TAA13 | 246.98 |  |  |  |  |
|  |  | TAA14 | 149.20 |  |  |  |  |
|  |  | TAA15 | 158.58 |  |  |  |  |
|  |  | TAA16 | 233.60 |  |  |  |  |
|  |  | TAA17 | 152.68 |  |  |  |  |
|  |  | TAA18 | 150.79 |  |  |  |  |
|  |  | mean | **201.02** |  |  |  |  |
|  |  | SD | **40.47** |  |  |  |  |
|  | SOD hipp |  |  |  |  |  |  |
|  | control |  | TAA |  | FIN |  | FIN+TAA |
| C1 | 125.10 | TAA1 | 151.56 | FIN1 | 160.48 | FIN+TAA1 | 148.14 |
| C2 | 122.67 | TAA2 | 170.94 | FIN2 | 124.43 | FIN+TAA2 | 132.93 |
| C3 | 111.06 | TAA3 | 160.88 | FIN3 | 133.59 | FIN+TAA3 | 102.07 |
| C4 | 126.50 | TAA4 | 103.93 | FIN4 | 137.22 | FIN+TAA4 | 134.82 |
| C5 | 110.47 | TAA5 | 127.78 | FIN5 | 179.23 | FIN+TAA5 | 132.04 |
| C6 | 102.19 | TAA6 | 114.21 | FIN6 | 152.40 | FIN+TAA6 | 160.15 |
| C7 | 115.26 | TAA7 | 166.16 | FIN7 | 130.97 | FIN+TAA7 | 139.51 |
| C8 | 129.71 | TAA8 | 116.31 | FIN8 | 147.11 | FIN+TAA8 | 146.82 |
| mean | 117.87 | TAA9 | 129.63 | mean | 145.68 | mean | 137.06 |
| SD | 9.59 | TAA10 | 138.25 | sd | 18.04 | sd | 17.02 |
|  |  | TAA11 | 165.81 |  |  |  |  |
|  |  | TAA12 | 169.20 |  |  |  |  |
|  |  | TAA13 | 123.96 |  |  |  |  |
|  |  | TAA14 | 122.23 |  |  |  |  |
|  |  | TAA15 | 125.10 |  |  |  |  |
|  |  | TAA16 | 176.84 |  |  |  |  |
|  |  | TAA17 | 101.96 |  |  |  |  |
|  |  | TAA18 | 135.01 |  |  |  |  |
|  |  | mean | **138.88** |  |  |  |  |
|  |  | SD | **24.40** |  |  |  |  |
|  | SOD thal |  |  |  |  |  |  |
|  | control |  | TAA |  | FIN |  | FIN+TAA |
| C1 | 195.03 | TAA1 | 128.61 | FIN1 | 127.62 | FIN+TAA1 | 170.87 |
| C2 | 147.76 | TAA2 | 155.44 | FIN2 | 139.53 | FIN+TAA2 | 137.94 |
| C3 | 136.05 | TAA3 | 170.73 | FIN3 | 107.60 | FIN+TAA3 | 142.50 |
| C4 | 127.79 | TAA4 | 134.62 | FIN4 | 127.32 | FIN+TAA4 | 162.12 |
| C5 | 155.28 | TAA5 | 132.56 | FIN5 | 104.10 | FIN+TAA5 | 134.37 |
| C6 | 142.68 | TAA6 | 169.82 | FIN6 | 122.44 | FIN+TAA6 | 137.99 |
| C7 | 150.54 | TAA7 | 136.64 | FIN7 | 150.23 | FIN+TAA7 | 171.17 |
| C8 | 161.77 | TAA8 | 135.05 | FIN8 | 147.08 | FIN+TAA8 | 177.57 |
| mean | 152.11 | TAA9 | 116.26 | mean | 128.24 | mean | 154.32 |
| SD | 20.37 | TAA10 | 127.80 | sd | 16.94 | sd | 17.85 |
|  |  | TAA11 | 131.97 |  |  |  |  |
|  |  | TAA12 | 136.19 |  |  |  |  |
|  |  | TAA13 | 158.76 |  |  |  |  |
|  |  | TAA14 | 153.79 |  |  |  |  |
|  |  | TAA15 | 144.93 |  |  |  |  |
|  |  | TAA16 | 143.31 |  |  |  |  |
|  |  | TAA17 | 153.07 |  |  |  |  |
|  |  | TAA18 | 119.62 |  |  |  |  |
|  |  | mean | **141.62** |  |  |  |  |
|  |  | SD | **15.77** |  |  |  |  |
|  | SOD nc |  |  |  |  |  |  |
|  | control |  | TAA |  | FIN |  | FIN+TAA |
| C1 | 88.48 | TAA1 | 170.65 | FIN1 | 135.87 | FIN+TAA1 | 139.21 |
| C2 | 105.05 | TAA2 | 77.09 | FIN2 | 115.59 | FIN+TAA2 | 102.27 |
| C3 | 92.36 | TAA3 | 91.86 | FIN3 | 71.56 | FIN+TAA3 | 114.02 |
| C4 | 87.54 | TAA4 | 161.82 | FIN4 | 80.55 | FIN+TAA4 | 147.35 |
| C5 | 112.12 | TAA5 | 105.15 | FIN5 | 71.69 | FIN+TAA5 | 123.65 |
| C6 | 103.96 | TAA6 | 97.76 | FIN6 | 126.79 | FIN+TAA6 | 114.74 |
| C7 | 100.57 | TAA7 | 96.96 | FIN7 | 128.55 | FIN+TAA7 | 163.94 |
| C8 | 74.15 | TAA8 | 118.23 | FIN8 | 85.70 | FIN+TAA8 | 131.39 |
| mean | 95.53 | TAA9 | 161.74 | mean | 102.03 | mean | 129.57 |
| SD | 12.21 | TAA10 | 128.91 | sd | 27.32 | sd | 20.13 |
|  |  | TAA11 | 121.20 |  |  |  |  |
|  |  | TAA12 | 103.33 |  |  |  |  |
|  |  | TAA13 | 117.02 |  |  |  |  |
|  |  | TAA14 | 112.90 |  |  |  |  |
|  |  | TAA15 | 103.05 |  |  |  |  |
|  |  | TAA16 | 104.55 |  |  |  |  |
|  |  | TAA17 | 188.17 |  |  |  |  |
|  |  | TAA18 | 107.92 |  |  |  |  |
|  |  | mean | **120.46** |  |  |  |  |
|  |  | SD | **30.36** |  |  |  |  |
|  | GSH cort |  |  |  |  |  |  |
|  | control |  | TAA |  | FIN |  | FIN+TAA |
| C1 | 57.11 | TAA1 | 135.90 | FIN1 | 32.07 | FIN+TAA1 | 110.98 |
| C2 | 30.50 | TAA2 | 142.30 | FIN2 | 44.39 | FIN+TAA2 | 77.24 |
| C3 | 29.51 | TAA3 | 132.73 | FIN3 | 34.86 | FIN+TAA3 | 56.38 |
| C4 | 53.47 | TAA4 | 66.03 | FIN4 | 38.20 | FIN+TAA4 | 51.02 |
| C5 | 57.38 | TAA5 | 99.27 | FIN5 | 41.29 | FIN+TAA5 | 132.16 |
| C6 | 32.51 | TAA6 | 133.41 | FIN6 | 47.10 | FIN+TAA6 | 79.25 |
| C7 | 31.38 | TAA7 | 111.78 | FIN7 | 44.77 | FIN+TAA7 | 100.63 |
| C8 | 59.98 | TAA8 | 78.72 | FIN8 | 45.91 | FIN+TAA8 | 84.50 |
| mean | 43.98 | TAA9 | 89.36 | mean | 41.07 | mean | 86.52 |
| SD | 14.04 | TAA10 | 93.44 | sd | 5.51 | sd | 27.23 |
|  |  | TAA11 | 149.56 |  |  |  |  |
|  |  | TAA12 | 73.42 |  |  |  |  |
|  |  | TAA13 | 61.74 |  |  |  |  |
|  |  | TAA14 | 134.80 |  |  |  |  |
|  |  | TAA15 | 81.63 |  |  |  |  |
|  |  | TAA16 | 51.10 |  |  |  |  |
|  |  | TAA17 | 133.41 |  |  |  |  |
|  |  | TAA18 | 108.61 |  |  |  |  |
|  |  | mean | **104.29** |  |  |  |  |
|  |  | SD | **31.24** |  |  |  |  |

|  | GSH hipp |  |  |  |  |  |  |
| --- | --- | --- | --- | --- | --- | --- | --- |
|  | control |  | TAA |  | FIN |  | FIN+TAA |
| C1 | 27.60 | TAA1 | 32.28 | FIN1 | 50.64 | FIN+TAA1 | 21.52 |
| C2 | 45.29 | TAA2 | 32.66 | FIN2 | 53.27 | FIN+TAA2 | 39.75 |
| C3 | 38.56 | TAA3 | 16.54 | FIN3 | 33.89 | FIN+TAA3 | 41.01 |
| C4 | 43.12 | TAA4 | 14.13 | FIN4 | 49.13 | FIN+TAA4 | 38.94 |
| C5 | 30.16 | TAA5 | 29.01 | FIN5 | 37.41 | FIN+TAA5 | 23.87 |
| C6 | 46.92 | TAA6 | 27.47 | FIN6 | 39.47 | FIN+TAA6 | 43.64 |
| C7 | 43.38 | TAA7 | 31.69 | FIN7 | 40.20 | FIN+TAA7 | 40.71 |
| C8 | 42.36 | TAA8 | 35.41 | FIN8 | 53.06 | FIN+TAA8 | 24.68 |
| mean | **39.67** | TAA9 | 14.20 | mean | **44.64** | mean | **34.26** |
| SD | **7.11** | TAA10 | 16.42 | sd | **7.71** | sd | **9.17** |
|  |  | TAA11 | 17.98 |  |  |  |  |
|  |  | TAA12 | 19.65 |  |  |  |  |
|  |  | TAA13 | 36.42 |  |  |  |  |
|  |  | TAA14 | 26.72 |  |  |  |  |
|  |  | TAA15 | 14.48 |  |  |  |  |
|  |  | TAA16 | 21.84 |  |  |  |  |
|  |  | TAA17 | 16.86 |  |  |  |  |
|  |  | TAA18 | 29.13 |  |  |  |  |
|  |  | mean | **24.05** |  |  |  |  |
|  |  | SD | **7.92** |  |  |  |  |
|  | GSH thal |  |  |  |  |  |  |
|  | control |  | TAA |  | FIN |  | FIN+TAA |
| C1 | 31.91 | TAA1 | 24.61 | FIN1 | 27.12 | FIN+TAA1 | 17.52 |
| C2 | 38.65 | TAA2 | 35.09 | FIN2 | 33.80 | FIN+TAA2 | 26.47 |
| C3 | 31.07 | TAA3 | 15.18 | FIN3 | 26.28 | FIN+TAA3 | 18.37 |
| C4 | 20.72 | TAA4 | 31.74 | FIN4 | 34.30 | FIN+TAA4 | 18.45 |
| C5 | 22.14 | TAA5 | 26.86 | FIN5 | 35.98 | FIN+TAA5 | 15.52 |
| C6 | 18.24 | TAA6 | 38.38 | FIN6 | 37.55 | FIN+TAA6 | 19.13 |
| C7 | 33.96 | TAA7 | 33.55 | FIN7 | 35.56 | FIN+TAA7 | 18.08 |
| C8 | 33.40 | TAA8 | 33.04 | FIN8 | 26.02 | FIN+TAA8 | 21.45 |
| mean | 28.76 | TAA9 | 31.84 | mean | 32.08 | mean | 19.37 |
| SD | 7.37 | TAA10 | 19.81 | sd | 4.78 | sd | 3.31 |
|  |  | TAA11 | 35.75 |  |  |  |  |
|  |  | TAA12 | 36.49 |  |  |  |  |
|  |  | TAA13 | 27.33 |  |  |  |  |
|  |  | TAA14 | 33.69 |  |  |  |  |
|  |  | TAA15 | 31.02 |  |  |  |  |
|  |  | TAA16 | 38.80 |  |  |  |  |
|  |  | TAA17 | 13.72 |  |  |  |  |
|  |  | TAA18 | 23.16 |  |  |  |  |
|  |  | mean | **29.45** |  |  |  |  |
|  |  | SD | **7.54** |  |  |  |  |
|  | GSH nc |  |  |  |  |  |  |
|  | control |  | TAA |  | FIN |  | FIN+TAA |
| C1 | 44.93 | TAA1 | 18.25 | FIN1 | 19.62 | FIN+TAA1 | 15.71 |
| C2 | 47.12 | TAA2 | 19.80 | FIN2 | 40.12 | FIN+TAA2 | 10.42 |
| C3 | 40.28 | TAA3 | 18.52 | FIN3 | 33.55 | FIN+TAA3 | 12.48 |
| C4 | 26.08 | TAA4 | 25.67 | FIN4 | 23.82 | FIN+TAA4 | 11.87 |
| C5 | 24.81 | TAA5 | 24.17 | FIN5 | 37.05 | FIN+TAA5 | 16.71 |
| C6 | 25.06 | TAA6 | 29.50 | FIN6 | 33.46 | FIN+TAA6 | 10.06 |
| C7 | 42.79 | TAA7 | 31.79 | FIN7 | 41.26 | FIN+TAA7 | 16.77 |
| C8 | 26.56 | TAA8 | 23.67 | FIN8 | 24.21 | FIN+TAA8 | 11.42 |
| mean | **34.70** | TAA9 | 19.61 | mean | **31.64** | mean | **13.18** |
| SD | **9.91** | TAA10 | 27.43 | sd | **8.12** | sd | **2.79** |
|  |  | TAA11 | 20.27 |  |  |  |  |
|  |  | TAA12 | 24.95 |  |  |  |  |
|  |  | TAA13 | 21.89 |  |  |  |  |
|  |  | TAA14 | 24.62 |  |  |  |  |
|  |  | TAA15 | 22.65 |  |  |  |  |
|  |  | TAA16 | 20.68 |  |  |  |  |
|  |  | TAA17 | 23.83 |  |  |  |  |
|  |  | TAA18 | 24.93 |  |  |  |  |
|  |  | mean | **23.46** |  |  |  |  |
|  |  | SD | **3.71** |  |  |  |  |
|  | GPx cort |  |  |  |  |  |  |
|  | control |  | TAA |  | FIN |  | FIN+TAA |
| C1 | 85.11 | TAA1 | 157.45 | FIN1 | 42.29 | FIN+TAA1 | 85.67 |
| C2 | 108.05 | TAA2 | 90.07 | FIN2 | 69.05 | FIN+TAA2 | 130.21 |
| C3 | 105.58 | TAA3 | 114.11 | FIN3 | 51.34 | FIN+TAA3 | 124.24 |
| C4 | 112.47 | TAA4 | 109.90 | FIN4 | 45.57 | FIN+TAA4 | 103.45 |
| C5 | 86.87 | TAA5 | 96.45 | FIN5 | 80.44 | FIN+TAA5 | 101.25 |
| C6 | 115.45 | TAA6 | 111.35 | FIN6 | 71.86 | FIN+TAA6 | 121.28 |
| C7 | 96.70 | TAA7 | 118.02 | FIN7 | 41.35 | FIN+TAA7 | 134.36 |
| C8 | 111.05 | TAA8 | 110.19 | FIN8 | 65.11 | FIN+TAA8 | 95.93 |
| mean | 102.66 | TAA9 | 103.61 | mean | 58.38 | mean | 112.05 |
| SD | 11.72 | TAA10 | 91.49 | sd | 15.07 | sd | 17.76 |
|  |  | TAA11 | 143.87 |  |  |  |  |
|  |  | TAA12 | 133.36 |  |  |  |  |
|  |  | TAA13 | 149.62 |  |  |  |  |
|  |  | TAA14 | 142.60 |  |  |  |  |
|  |  | TAA15 | 135.26 |  |  |  |  |
|  |  | TAA16 | 114.58 |  |  |  |  |
|  |  | TAA17 | 159.85 |  |  |  |  |
|  |  | TAA18 | 138.35 |  |  |  |  |
|  |  | mean | **123.34** |  |  |  |  |
|  |  | SD | **22.19** |  |  |  |  |
|  | GPx hipp |  |  |  |  |  |  |
|  | control |  | TAA |  | FIN |  | FIN+TAA |
| C1 | 90.49 | TAA1 | 83.04 | FIN1 | 54.59 | FIN+TAA1 | 74.07 |
| C2 | 43.79 | TAA2 | 106.18 | FIN2 | 39.90 | FIN+TAA2 | 68.64 |
| C3 | 49.44 | TAA3 | 66.16 | FIN3 | 63.87 | FIN+TAA3 | 45.18 |
| C4 | 89.05 | TAA4 | 52.01 | FIN4 | 75.91 | FIN+TAA4 | 49.73 |
| C5 | 69.58 | TAA5 | 69.14 | FIN5 | 91.43 | FIN+TAA5 | 106.89 |
| C6 | 51.51 | TAA6 | 94.68 | FIN6 | 51.97 | FIN+TAA6 | 89.31 |
| C7 | 75.01 | TAA7 | 55.81 | FIN7 | 93.09 | FIN+TAA7 | 62.66 |
| C8 | 84.15 | TAA8 | 82.78 | FIN8 | 56.45 | FIN+TAA8 | 93.25 |
| mean | 69.13 | TAA9 | 97.56 | mean | 65.90 | mean | 73.72 |
| SD | 18.72 | TAA10 | 72.62 | sd | 19.21 | sd | 21.59 |
|  |  | TAA11 | 83.30 |  |  |  |  |
|  |  | TAA12 | 107.79 |  |  |  |  |
|  |  | TAA13 | 82.80 |  |  |  |  |
|  |  | TAA14 | 58.78 |  |  |  |  |
|  |  | TAA15 | 84.82 |  |  |  |  |
|  |  | TAA16 | 91.74 |  |  |  |  |
|  |  | TAA17 | 52.02 |  |  |  |  |
|  |  | TAA18 | 55.30 |  |  |  |  |
|  |  | mean | **77.58** |  |  |  |  |
|  |  | SD | **18.23** |  |  |  |  |

|  | GPx thal |  |  |  |  |  |  |
| --- | --- | --- | --- | --- | --- | --- | --- |
|  | control |  | TAA |  | FIN |  | FIN+TAA |
| C1 | 181.66 | TAA1 | 305.69 | FIN1 | 60.63 | FIN+TAA1 | 164.09 |
| C2 | 178.92 | TAA2 | 275.20 | FIN2 | 35.89 | FIN+TAA2 | 155.78 |
| C3 | 148.89 | TAA3 | 159.42 | FIN3 | 57.59 | FIN+TAA3 | 211.49 |
| C4 | 142.88 | TAA4 | 181.57 | FIN4 | 54.00 | FIN+TAA4 | 197.08 |
| C5 | 139.54 | TAA5 | 267.29 | FIN5 | 41.05 | FIN+TAA5 | 206.70 |
| C6 | 184.74 | TAA6 | 279.05 | FIN6 | 56.40 | FIN+TAA6 | 170.43 |
| C7 | 144.78 | TAA7 | 227.66 | FIN7 | 31.06 | FIN+TAA7 | 159.17 |
| C8 | 179.47 | TAA8 | 207.02 | FIN8 | 56.73 | FIN+TAA8 | 148.95 |
| mean | **162.61** | TAA9 | 200.77 | mean | **49.17** | mean | **176.71** |
| SD | **20.11** | TAA10 | 188.72 | sd | **11.37** | sd | **24.61** |
|  |  | TAA11 | 194.71 |  |  |  |  |
|  |  | TAA12 | 211.75 |  |  |  |  |
|  |  | TAA13 | 272.80 |  |  |  |  |
|  |  | TAA14 | 245.43 |  |  |  |  |
|  |  | TAA15 | 190.11 |  |  |  |  |
|  |  | TAA16 | 343.20 |  |  |  |  |
|  |  | TAA17 | 216.06 |  |  |  |  |
|  |  | TAA18 | 265.23 |  |  |  |  |
|  |  | mean | **235.09** |  |  |  |  |
|  |  | SD | **49.12** |  |  |  |  |
|  | GPx nc |  |  |  |  |  |  |
|  | control |  | TAA |  | FIN |  | FIN+TAA |
| C1 | 52.53 | TAA1 | 138.40 | FIN1 | 88.42 | FIN+TAA1 | 63.16 |
| C2 | 48.08 | TAA2 | 89.33 | FIN2 | 68.26 | FIN+TAA2 | 73.81 |
| C3 | 70.03 | TAA3 | 141.70 | FIN3 | 63.84 | FIN+TAA3 | 62.32 |
| C4 | 65.61 | TAA4 | 112.74 | FIN4 | 63.17 | FIN+TAA4 | 46.88 |
| C5 | 73.35 | TAA5 | 100.95 | FIN5 | 64.83 | FIN+TAA5 | 43.90 |
| C6 | 76.30 | TAA6 | 111.70 | FIN6 | 62.57 | FIN+TAA6 | 76.48 |
| C7 | 65.54 | TAA7 | 112.40 | FIN7 | 85.61 | FIN+TAA7 | 53.50 |
| C8 | 91.36 | TAA8 | 133.33 | FIN8 | 79.42 | FIN+TAA8 | 42.50 |
| mean | 67.85 | TAA9 | 102.19 | mean | 72.01 | mean | 57.82 |
| SD | 13.60 | TAA10 | 86.31 | sd | 10.75 | sd | 13.20 |
|  |  | TAA11 | 120.15 |  |  |  |  |
|  |  | TAA12 | 98.96 |  |  |  |  |
|  |  | TAA13 | 109.56 |  |  |  |  |
|  |  | TAA14 | 94.32 |  |  |  |  |
|  |  | TAA15 | 89.76 |  |  |  |  |
|  |  | TAA16 | 137.36 |  |  |  |  |
|  |  | TAA17 | 102.35 |  |  |  |  |
|  |  | TAA18 | 91.52 |  |  |  |  |
|  |  | mean | **109.61** |  |  |  |  |
|  |  | SD | **18.03** |  |  |  |  |
|  | GR cort |  |  |  |  |  |  |
|  | control |  | TAA |  | FIN |  | FIN+TAA |
| C1 | 8.42 | TAA1 | 7.65 | FIN1 | 7.94 | FIN+TAA1 | 7.40 |
| C2 | 5.77 | TAA2 | 11.06 | FIN2 | 7.74 | FIN+TAA2 | 5.60 |
| C3 | 4.81 | TAA3 | 8.57 | FIN3 | 4.17 | FIN+TAA3 | 9.92 |
| C4 | 6.39 | TAA4 | 10.71 | FIN4 | 8.35 | FIN+TAA4 | 10.45 |
| C5 | 4.12 | TAA5 | 11.32 | FIN5 | 7.93 | FIN+TAA5 | 3.63 |
| C6 | 4.60 | TAA6 | 7.16 | FIN6 | 8.05 | FIN+TAA6 | 4.84 |
| C7 | 5.06 | TAA7 | 6.80 | FIN7 | 3.93 | FIN+TAA7 | 4.22 |
| C8 | 10.66 | TAA8 | 7.29 | FIN8 | 8.37 | FIN+TAA8 | 4.87 |
| mean | 6.23 | TAA9 | 10.53 | mean | 7.06 | mean | 6.37 |
| SD | 2.24 | TAA10 | 10.45 | sd | 1.87 | sd | 2.61 |
|  |  | TAA11 | 9.44 |  |  |  |  |
|  |  | TAA12 | 12.32 |  |  |  |  |
|  |  | TAA13 | 9.78 |  |  |  |  |
|  |  | TAA14 | 8.90 |  |  |  |  |
|  |  | TAA15 | 11.39 |  |  |  |  |
|  |  | TAA16 | 10.01 |  |  |  |  |
|  |  | TAA17 | 10.44 |  |  |  |  |
|  |  | TAA18 | 11.60 |  |  |  |  |
|  |  | mean | **9.74** |  |  |  |  |
|  |  | SD | **1.67** |  |  |  |  |
|  | GR hipp |  |  |  |  |  |  |
|  | control |  | TAA |  | FIN |  | FIN+TAA |
| C1 | 11.31 | TAA1 | 9.25 | FIN1 | 11.92 | FIN+TAA1 | 10.48 |
| C2 | 9.81 | TAA2 | 12.44 | FIN2 | 9.85 | FIN+TAA2 | 12.76 |
| C3 | 12.98 | TAA3 | 12.93 | FIN3 | 12.82 | FIN+TAA3 | 10.99 |
| C4 | 10.09 | TAA4 | 13.87 | FIN4 | 8.24 | FIN+TAA4 | 9.56 |
| C5 | 9.00 | TAA5 | 12.88 | FIN5 | 11.98 | FIN+TAA5 | 9.92 |
| C6 | 8.73 | TAA6 | 10.04 | FIN6 | 8.63 | FIN+TAA6 | 11.01 |
| C7 | 12.69 | TAA7 | 13.90 | FIN7 | 8.25 | FIN+TAA7 | 10.12 |
| C8 | 10.89 | TAA8 | 12.87 | FIN8 | 7.67 | FIN+TAA8 | 10.05 |
| mean | 10.69 | TAA9 | 16.39 | mean | 9.92 | mean | 10.61 |
| SD | 1.58 | TAA10 | 12.21 | sd | 2.04 | sd | 1.01 |
|  |  | TAA11 | 14.30 |  |  |  |  |
|  |  | TAA12 | 14.44 |  |  |  |  |
|  |  | TAA13 | 14.41 |  |  |  |  |
|  |  | TAA14 | 16.69 |  |  |  |  |
|  |  | TAA15 | 11.98 |  |  |  |  |
|  |  | TAA16 | 14.26 |  |  |  |  |
|  |  | TAA17 | 11.16 |  |  |  |  |
|  |  | TAA18 | 12.52 |  |  |  |  |
|  |  | mean | **13.14** |  |  |  |  |
|  |  | SD | **1.91** |  |  |  |  |
|  | GR thal |  |  |  |  |  |  |
|  | control |  | TAA |  | FIN |  | FIN+TAA |
| C1 | 8.63 | TAA1 | 15.03 | FIN1 | 9.30 | FIN+TAA1 | 10.51 |
| C2 | 8.57 | TAA2 | 12.05 | FIN2 | 9.49 | FIN+TAA2 | 3.70 |
| C3 | 8.88 | TAA3 | 9.57 | FIN3 | 7.79 | FIN+TAA3 | 6.25 |
| C4 | 10.15 | TAA4 | 12.07 | FIN4 | 9.75 | FIN+TAA4 | 10.16 |
| C5 | 10.71 | TAA5 | 12.04 | FIN5 | 8.18 | FIN+TAA5 | 6.49 |
| C6 | 7.99 | TAA6 | 8.40 | FIN6 | 8.71 | FIN+TAA6 | 9.99 |
| C7 | 8.67 | TAA7 | 17.07 | FIN7 | 11.02 | FIN+TAA7 | 7.31 |
| C8 | 8.01 | TAA8 | 12.49 | FIN8 | 10.54 | FIN+TAA8 | 5.16 |
| mean | 8.95 | TAA9 | 13.25 | mean | 9.35 | mean | 7.44 |
| SD | 0.98 | TAA10 | 10.61 | sd | 1.11 | sd | 2.53 |
|  |  | TAA11 | 13.14 |  |  |  |  |
|  |  | TAA12 | 6.92 |  |  |  |  |
|  |  | TAA13 | 17.08 |  |  |  |  |
|  |  | TAA14 | 6.07 |  |  |  |  |
|  |  | TAA15 | 12.08 |  |  |  |  |
|  |  | TAA16 | 12.28 |  |  |  |  |
|  |  | TAA17 | 8.82 |  |  |  |  |
|  |  | TAA18 | 9.25 |  |  |  |  |
|  |  | mean | **11.57** |  |  |  |  |
|  |  | SD | **3.07** |  |  |  |  |
|  | GR nc |  |  |  |  |  |  |
|  | control |  | TAA |  | FIN |  | FIN+TAA |
| C1 | 12.06 | TAA1 | 20.24 | FIN1 | 8.29 | FIN+TAA1 | 5.99 |
| C2 | 10.45 | TAA2 | 18.62 | FIN2 | 8.98 | FIN+TAA2 | 12.15 |
| C3 | 10.75 | TAA3 | 19.91 | FIN3 | 11.44 | FIN+TAA3 | 13.01 |
| C4 | 7.40 | TAA4 | 12.34 | FIN4 | 12.07 | FIN+TAA4 | 6.96 |
| C5 | 6.55 | TAA5 | 10.54 | FIN5 | 11.99 | FIN+TAA5 | 6.38 |
| C6 | 13.83 | TAA6 | 18.32 | FIN6 | 8.26 | FIN+TAA6 | 10.52 |
| C7 | 8.56 | TAA7 | 10.88 | FIN7 | 8.76 | FIN+TAA7 | 12.99 |
| C8 | 13.23 | TAA8 | 7.47 | FIN8 | 9.87 | FIN+TAA8 | 11.57 |
| mean | 10.35 | TAA9 | 14.67 | mean | 9.96 | mean | 9.95 |
| SD | 2.67 | TAA10 | 12.66 | sd | 1.64 | sd | 3.02 |
|  |  | TAA11 | 14.54 |  |  |  |  |
|  |  | TAA12 | 16.72 |  |  |  |  |
|  |  | TAA13 | 15.46 |  |  |  |  |
|  |  | TAA14 | 17.53 |  |  |  |  |
|  |  | TAA15 | 16.51 |  |  |  |  |
|  |  | TAA16 | 13.07 |  |  |  |  |
|  |  | TAA17 | 12.06 |  |  |  |  |
|  |  | TAA18 | 13.19 |  |  |  |  |
|  |  | mean | **14.71** |  |  |  |  |
|  |  | SD | **3.50** |  |  |  |  |
|  | cat cort |  |  |  |  |  |  |
|  | control |  | TAA |  | FIN |  | FIN+TAA |
| C1 | 11.14 | TAA1 | 7.68 | FIN1 | 11.31 | FIN+TAA1 | 8.34 |
| C2 | 11.93 | TAA2 | 9.20 | FIN2 | 9.10 | FIN+TAA2 | 8.64 |
| C3 | 8.31 | TAA3 | 6.18 | FIN3 | 8.33 | FIN+TAA3 | 8.03 |
| C4 | 8.52 | TAA4 | 4.71 | FIN4 | 8.73 | FIN+TAA4 | 9.35 |
| C5 | 12.02 | TAA5 | 5.85 | FIN5 | 9.26 | FIN+TAA5 | 11.31 |
| C6 | 11.54 | TAA6 | 8.89 | FIN6 | 11.78 | FIN+TAA6 | 10.12 |
| C7 | 10.64 | TAA7 | 9.34 | FIN7 | 9.41 | FIN+TAA7 | 9.59 |
| C8 | 7.07 | TAA8 | 7.70 | FIN8 | 9.25 | FIN+TAA8 | 8.52 |
| mean | 10.15 | TAA9 | 4.83 | mean | 9.65 | mean | 9.24 |
| SD | 1.90 | TAA10 | 5.72 | sd | 1.23 | sd | 1.09 |
|  |  | TAA11 | 5.63 |  |  |  |  |
|  |  | TAA12 | 6.54 |  |  |  |  |
|  |  | TAA13 | 10.17 |  |  |  |  |
|  |  | TAA14 | 7.95 |  |  |  |  |
|  |  | TAA15 | 7.24 |  |  |  |  |
|  |  | TAA16 | 5.05 |  |  |  |  |
|  |  | TAA17 | 4.79 |  |  |  |  |
|  |  | TAA18 | 5.09 |  |  |  |  |
|  |  | mean | **6.81** |  |  |  |  |
|  |  | SD | **1.77** |  |  |  |  |
|  | cat hipp |  |  |  |  |  |  |
|  | control |  | TAA |  | FIN |  | FIN+TAA |
| C1 | 5.92 | TAA1 | 4.51 | FIN1 | 5.15 | FIN+TAA1 | 3.05 |
| C2 | 3.98 | TAA2 | 1.45 | FIN2 | 7.89 | FIN+TAA2 | 3.07 |
| C3 | 5.10 | TAA3 | 3.06 | FIN3 | 3.97 | FIN+TAA3 | 1.85 |
| C4 | 7.60 | TAA4 | 4.38 | FIN4 | 5.62 | FIN+TAA4 | 1.16 |
| C5 | 8.40 | TAA5 | 2.13 | FIN5 | 5.15 | FIN+TAA5 | 2.84 |
| C6 | 3.60 | TAA6 | 2.77 | FIN6 | 7.12 | FIN+TAA6 | 2.96 |
| C7 | 8.70 | TAA7 | 3.48 | FIN7 | 7.29 | FIN+TAA7 | 1.37 |
| C8 | 5.78 | TAA8 | 2.78 | FIN8 | 4.30 | FIN+TAA8 | 4.02 |
| mean | 6.13 | TAA9 | 4.50 | mean | 5.81 | mean | 2.54 |
| SD | 1.93 | TAA10 | 2.93 | sd | 1.45 | sd | 0.98 |
|  |  | TAA11 | 2.27 |  |  |  |  |
|  |  | TAA12 | 2.58 |  |  |  |  |
|  |  | TAA13 | 1.61 |  |  |  |  |
|  |  | TAA14 | 2.86 |  |  |  |  |
|  |  | TAA15 | 2.67 |  |  |  |  |
|  |  | TAA16 | 3.38 |  |  |  |  |
|  |  | TAA17 | 3.77 |  |  |  |  |
|  |  | TAA18 | 3.92 |  |  |  |  |
|  |  | mean | **3.06** |  |  |  |  |
|  |  | SD | **0.92** |  |  |  |  |
|  | cat thal |  |  |  |  |  |  |
|  | control |  | TAA |  | FIN |  | FIN+TAA |
| C1 | 7.48 | TAA1 | 0.97 | FIN1 | 6.12 | FIN+TAA1 | 2.19 |
| C2 | 3.40 | TAA2 | 2.08 | FIN2 | 4.90 | FIN+TAA2 | 2.47 |
| C3 | 5.05 | TAA3 | 2.39 | FIN3 | 5.00 | FIN+TAA3 | 3.94 |
| C4 | 7.96 | TAA4 | 1.65 | FIN4 | 4.76 | FIN+TAA4 | 1.74 |
| C5 | 4.34 | TAA5 | 1.84 | FIN5 | 4.98 | FIN+TAA5 | 1.83 |
| C6 | 6.42 | TAA6 | 2.17 | FIN6 | 5.64 | FIN+TAA6 | 1.59 |
| C7 | 4.99 | TAA7 | 1.10 | FIN7 | 8.67 | FIN+TAA7 | 2.80 |
| C8 | 4.62 | TAA8 | 1.79 | FIN8 | 5.40 | FIN+TAA8 | 3.63 |
| mean | 5.53 | TAA9 | 2.69 | mean | 5.68 | mean | 2.52 |
| SD | 1.60 | TAA10 | 1.30 | sd | 1.29 | sd | 0.88 |
|  |  | TAA11 | 1.71 |  |  |  |  |
|  |  | TAA12 | 0.91 |  |  |  |  |
|  |  | TAA13 | 1.99 |  |  |  |  |
|  |  | TAA14 | 2.72 |  |  |  |  |
|  |  | TAA15 | 2.76 |  |  |  |  |
|  |  | TAA16 | 2.60 |  |  |  |  |
|  |  | TAA17 | 2.58 |  |  |  |  |
|  |  | TAA18 | 1.97 |  |  |  |  |
|  |  | mean | **1.96** |  |  |  |  |
|  |  | SD | **0.61** |  |  |  |  |

|  | cat nc |  | |  |  |  |  | |  | |
| --- | --- | --- | --- | --- | --- | --- | --- | --- | --- | --- |
|  | control |  | | TAA |  | FIN |  | | FIN+TAA | |
| C1 | 3.30 | TAA1 | | 3.50 | FIN1 | 6.05 | FIN+TAA1 | | 4.80 | |
| C2 | 2.25 | TAA2 | | 2.92 | FIN2 | 5.24 | FIN+TAA2 | | 2.82 | |
| C3 | 6.84 | TAA3 | | 2.09 | FIN3 | 4.85 | FIN+TAA3 | | 2.57 | |
| C4 | 3.49 | TAA4 | | 3.25 | FIN4 | 2.39 | FIN+TAA4 | | 3.36 | |
| C5 | 6.11 | TAA5 | | 3.16 | FIN5 | 2.01 | FIN+TAA5 | | 3.30 | |
| C6 | 3.32 | TAA6 | | 3.58 | FIN6 | 3.73 | FIN+TAA6 | | 2.94 | |
| C7 | 2.51 | TAA7 | | 3.29 | FIN7 | 6.78 | FIN+TAA7 | | 3.07 | |
| C8 | 5.38 | TAA8 | | 2.58 | FIN8 | 4.29 | FIN+TAA8 | | 4.64 | |
| mean | **4.15** | TAA9 | | 3.22 | mean | **4.42** | mean | | **3.44** | |
| SD | **1.72** | TAA10 | | 3.49 | sd | **1.67** | sd | | **0.83** | |
|  |  | TAA11 | | 3.33 |  |  |  | |  | |
|  |  | TAA12 | | 3.28 |  |  |  | |  | |
|  |  | TAA13 | | 2.55 |  |  |  | |  | |
|  |  | TAA14 | | 2.66 |  |  |  | |  | |
|  |  | TAA15 | | 3.34 |  |  |  | |  | |
|  |  | TAA16 | | 1.41 |  |  |  | |  | |
|  |  | TAA17 | | 3.16 |  |  |  | |  | |
|  |  | TAA18 | | 2.07 |  |  |  | |  | |
|  |  | mean | | **2.94** |  |  |  | |  | |
|  |  | SD | | **0.60** |  |  |  | |  | |
|  | AchE cort |  | |  |  |  |  | |  | |
|  | control |  | | TAA |  | FIN |  | | FIN+TAA | |
| C1 | 5.71 | TAA1 | | 3.65 | FIN1 | 6.25 | FIN+TAA1 | | 6.95 | |
| C2 | 5.49 | TAA2 | | 6.55 | FIN2 | 10.92 | FIN+TAA2 | | 7.11 | |
| C3 | 5.17 | TAA3 | | 5.39 | FIN3 | 12.26 | FIN+TAA3 | | 7.14 | |
| C4 | 6.44 | TAA4 | | 5.15 | FIN4 | 10.39 | FIN+TAA4 | | 6.47 | |
| C5 | 5.91 | TAA5 | | 8.62 | FIN5 | 7.86 | FIN+TAA5 | | 7.16 | |
| C6 | 5.29 | TAA6 | | 5.40 | FIN6 | 8.96 | FIN+TAA6 | | 6.37 | |
| C7 | 6.72 | TAA7 | | 5.14 | FIN7 | 8.46 | FIN+TAA7 | | 6.08 | |
| C8 | 5.34 | TAA8 | | 8.22 | FIN8 | 11.12 | FIN+TAA8 | | 6.30 | |
| mean | 5.76 | TAA9 | | 4.40 | mean | 9.53 | mean | | 6.70 | |
| SD | 0.56 | TAA10 | | 7.49 | sd | 1.99 | sd | | 0.44 | |
|  |  | TAA11 | | 4.38 |  |  |  | |  | |
|  |  | TAA12 | | 7.41 |  |  |  | |  | |
|  |  | TAA13 | | 9.06 |  |  |  | |  | |
|  |  | TAA14 | | 5.62 |  |  |  | |  | |
|  |  | TAA15 | | 6.58 |  |  |  | |  | |
|  |  | TAA16 | | 5.12 |  |  |  | |  | |
|  |  | TAA17 | | 8.13 |  |  |  | |  | |
|  |  | TAA18 | | 9.25 |  |  |  | |  | |
|  |  | mean | | **6.42** |  |  |  | |  | |
|  |  | SD | | **1.74** |  |  |  | |  | |
|  | AchE hipp | | |  |  |  |  |  | |  |
|  | control | |  | TAA |  | FIN |  | FIN+TAA | |  |
| C1 | 10.08 | | TAA1 | 10.81 | FIN1 | 19.19 | FIN+TAA1 | 10.23 | |  |
| C2 | 11.26 | | TAA2 | 13.81 | FIN2 | 16.37 | FIN+TAA2 | 11.07 | |  |
| C3 | 12.65 | | TAA3 | 15.49 | FIN3 | 15.03 | FIN+TAA3 | 13.18 | |  |
| C4 | 12.54 | | TAA4 | 10.35 | FIN4 | 20.05 | FIN+TAA4 | 11.50 | |  |
| C5 | 13.31 | | TAA5 | 12.49 | FIN5 | 14.52 | FIN+TAA5 | 9.83 | |  |
| C6 | 13.22 | | TAA6 | 15.65 | FIN6 | 20.99 | FIN+TAA6 | 10.45 | |  |
| C7 | 11.67 | | TAA7 | 11.83 | FIN7 | 19.53 | FIN+TAA7 | 15.85 | |  |
| C8 | 13.14 | | TAA8 | 15.16 | FIN8 | 13.80 | FIN+TAA8 | 14.22 | |  |
| mean | **12.23** | | TAA9 | 14.83 | mean | **17.44** | mean | **12.04** | |  |
| SD | **1.14** | | TAA10 | 9.23 | sd | **2.82** | sd | **2.15** | |  |
|  |  | | TAA11 | 8.95 |  |  |  |  | |  |
|  |  | | TAA12 | 8.36 |  |  |  |  | |  |
|  |  | | TAA13 | 17.61 |  |  |  |  | |  |
|  |  | | TAA14 | 15.87 |  |  |  |  | |  |
|  |  | | TAA15 | 9.88 |  |  |  |  | |  |
|  |  | | TAA16 | 14.81 |  |  |  |  | |  |
|  |  | | TAA17 | 13.94 |  |  |  |  | |  |
|  |  | | TAA18 | 8.09 |  |  |  |  | |  |
|  |  | | mean | **12.62** |  |  |  |  | |  |
|  |  | | SD | **2.99** |  |  |  |  | |  |
|  | AchE thal | |  |  |  |  |  |  | |  |
|  | control | |  | TAA |  | FIN |  | FIN+TAA | |  |
| C1 | 20.57 | | TAA1 | 24.09 | FIN1 | 20.83 | FIN+TAA1 | 14.73 | |  |
| C2 | 22.10 | | TAA2 | 29.75 | FIN2 | 20.95 | FIN+TAA2 | 19.50 | |  |
| C3 | 15.50 | | TAA3 | 19.47 | FIN3 | 30.55 | FIN+TAA3 | 18.22 | |  |
| C4 | 21.23 | | TAA4 | 25.92 | FIN4 | 27.90 | FIN+TAA4 | 14.24 | |  |
| C5 | 23.00 | | TAA5 | 20.07 | FIN5 | 23.29 | FIN+TAA5 | 17.81 | |  |
| C6 | 22.03 | | TAA6 | 27.67 | FIN6 | 31.09 | FIN+TAA6 | 14.88 | |  |
| C7 | 20.68 | | TAA7 | 27.54 | FIN7 | 22.78 | FIN+TAA7 | 18.39 | |  |
| C8 | 17.33 | | TAA8 | 21.11 | FIN8 | 25.00 | FIN+TAA8 | 19.15 | |  |
| mean | 20.31 | | TAA9 | 22.51 | mean | 25.30 | mean | 17.12 | |  |
| SD | 2.58 | | TAA10 | 19.58 | sd | 4.09 | sd | 2.14 | |  |
|  |  | | TAA11 | 23.32 |  |  |  |  | |  |
|  |  | | TAA12 | 21.28 |  |  |  |  | |  |
|  |  | | TAA13 | 30.11 |  |  |  |  | |  |
|  |  | | TAA14 | 23.54 |  |  |  |  | |  |
|  |  | | TAA15 | 25.49 |  |  |  |  | |  |
|  |  | | TAA16 | 24.84 |  |  |  |  | |  |
|  |  | | TAA17 | 21.49 |  |  |  |  | |  |
|  |  | | TAA18 | 27.16 |  |  |  |  | |  |
|  |  | | mean | **24.16** |  |  |  |  | |  |
|  |  | | SD | **3.37** |  |  |  |  | |  |
|  | AchE nc | |  |  |  |  |  |  | |  |
|  | control | |  | **TAA** |  | FIN |  | FIN+TAA | |  |
| C1 | 41.11 | | TAA1 | **34.70** | FIN1 | 33.08 | FIN+TAA1 | 41.62 | |  |
| C2 | 30.86 | | TAA2 | **71.32** | FIN2 | 55.36 | FIN+TAA2 | 46.93 | |  |
| C3 | 44.62 | | TAA3 | **39.37** | FIN3 | 57.14 | FIN+TAA3 | 23.58 | |  |
| C4 | 32.34 | | TAA4 | **42.09** | FIN4 | 61.19 | FIN+TAA4 | 24.01 | |  |
| C5 | 41.51 | | TAA5 | **70.45** | FIN5 | 39.29 | FIN+TAA5 | 38.54 | |  |
| C6 | 45.74 | | TAA6 | **62.33** | FIN6 | 50.32 | FIN+TAA6 | 45.95 | |  |
| C7 | 40.03 | | TAA7 | **64.24** | FIN7 | 38.85 | FIN+TAA7 | 44.49 | |  |
| C8 | 29.42 | | TAA8 | **65.53** | FIN8 | 56.57 | FIN+TAA8 | 28.82 | |  |
| mean | 38.20 | | TAA9 | **38.52** | mean | 48.98 | mean | 36.74 | |  |
| SD | 6.39 | | TAA10 | **37.60** | sd | 10.45 | sd | 9.81 | |  |
|  |  | | TAA11 | **51.87** |  |  |  |  | |  |
|  |  | | TAA12 | **37.87** |  |  |  |  | |  |
|  |  | | TAA13 | **51.18** |  |  |  |  | |  |
|  |  | | TAA14 | **71.65** |  |  |  |  | |  |
|  |  | | TAA15 | **71.03** |  |  |  |  | |  |
|  |  | | TAA16 | **42.44** |  |  |  |  | |  |
|  |  | | TAA17 | **52.29** |  |  |  |  | |  |
|  |  | | TAA18 | **69.27** |  |  |  |  | |  |
|  |  | | mean | **54.10** |  |  |  |  | |  |
|  |  | | SD | **14.06** |  |  |  |  | |  |
